# Cryptic population structure reveals low dispersal in Iberian wolves

**DOI:** 10.1038/s41598-018-32369-3

**Published:** 2018-09-20

**Authors:** Pedro Silva, José Vicente López-Bao, Luis Llaneza, Francisco Álvares, Susana Lopes, Juan Carlos Blanco, Yolanda Cortés, Emilio García, Vicente Palacios, Helena Rio-Maior, Nuno Ferrand, Raquel Godinho

**Affiliations:** 1 CIBIO/InBio - Centro de Investigação em Biodiversidade e Recursos Genéticos, Universidade do Porto, Campus de Vairão, 4485-661 Vairão, Portugal; 2 Departamento de Biologia, Faculdade de Ciências, Universidade do Porto, 4169-007 Porto, Portugal; 3 Research Unit of Biodiversity (UO/CSIC/PA), University of Oviedo, 33600 Mieres, Spain; 4 A.RE.NA, Asesores en Recursos Naturales, S.L 27003 Lugo, Spain; 5 Proyecto Lobo CBC, C/Daoíz, 28004 Madrid, Spain; 6 Department of Zoology, Faculty of Sciences, University of Johannesburg, Auckland Park, 2006 South Africa

## Abstract

Highly mobile mammalian carnivores are expected to have the capability to maintain high levels of gene flow across large geographic scales. Nonetheless, surprising levels of genetic structure have been found in many such populations. We combined genetic and spatial behavioural information from wolves (*Canis lupus*) in the Iberian Peninsula (Western Europe) during the last two decades to present a particular case of low dispersal levels in a large carnivore population persisting in human-dominated landscapes. We found an exceptionally reticulated pattern of cryptic population structure emerging at two hierarchical levels, in which four or eleven meaningful genetic clusters can be recognized, respectively. These clusters were characterized by moderate-high levels of differentiation (average pairwise F_ST_ = 0.09–0.19), low levels of admixture and varying degrees of genetic diversity. The number of dispersers identified among the 11 clusters was very low (<4% out of 218 wolves). Spatial information of tracked wolves further confirmed the geographical genetic patterns (only 2 out of 85 collared wolves overlapped with more than one genetic cluster). The high levels of genetic structure in this population may be determined by the recent demographic history of this population, among other factors. The identification of meaningful genetic clusters has implications for the delineation of conservation units and, consequently, on the conservation and management actions for Iberian wolves.

## Introduction

The rates at which populations exchange genes (i.e., gene flow) is one of the main driving forces determining the scale and magnitude of population genetic differentiation. It is expected that high rates of gene flow will lead to reduced spatial genetic structure at small scales, although isolation-by-distance patterns can emerge with increasing geographical distances, even in the absence of barriers to dispersal^1^. Many mammalian carnivores can disperse over very large distances and occupy a great variety of habitats. It is therefore expected that they have the potential for maintaining high rates of gene flow and consequently reduced genetic differentiation across large parts of their ranges^2–6^. However, recent studies have shown that continuous populations of these species can also exhibit notable levels of population genetic structure at different spatial scales, which cannot be explained by past or current barriers to dispersal alone^7–10^.

Traditionally, population genetic structure has been considered the outcome of well-defined behavioural traits (such as in the establishment of colonies^11^), spatial constraints (such as topographical or human-made barriers^12,13^), or historical factors (such as past range restrictions^14^). Other, human-related factors can also influence patterns of genetic structure. For example, hunting pressure has been observed to influence population structure in mountain lions *(Puma concolor)*^15^ and grey wolves (*Canis lupus*)^10^. However, recent studies have brought attention to less well-understood behavioural mechanisms promoting genetic population division by limiting dispersal, such as dietary specialization, natal habitat-biased dispersal and territoriality^8,9,16–21^. Wolves are an illustrative example of a species that is territorial, widely distributed, and moves across large distances^22^. Wolf dispersal distance is thought to be affected by population density and the probability of finding a mate^23–25^. New wolf packs can also be established on the edges of the natal territory of one of the founding individuals, leading to familial ties between geographically close groups^25^. Consequently, genetic clusters consisting of related individuals can emerge in wolf populations, although the level of genetic differentiation will depend on the rates of inter-pack gene flow^26^. Despite their high dispersal capabilities, wolf populations can nonetheless present low levels of gene flow and short dispersal distances, leading to genetic structure at small spatial scales and genetically differentiated groups that behave like a meta-population^3,26–29^.

In many populations, discrete and geographically coherent groupings of genetically similar individuals (clusters) have been identified even in the absence of gaps in the local distribution and/or of physical barriers to movement, and often without evident phenotypic distinction^30^ (cryptic population structure – CPS). The existence of these cryptic clusters is of great interest in terms of the behavioural and social processes they can reflect, their potential role in ecological and evolutionary processes, and ultimately their consequences for conservation and population management. Connectivity among clusters will have an effect on subpopulation growth rates and, consequently, may give rise to source-sink dynamics among cryptic clusters^15^. The combined use of behavioural data, such as spatial information from tagged animals, in addition to assignment procedures and estimates of differentiation from genetic data, has allowed the assessment of CPS patterns on a fine spatial scale and how individual clusters are spatially organized^13,30,31^.

The North-western Iberian wolf population, shared between Spain and Portugal, represents the largest wolf population in Western Europe, and is currently isolated from the remaining European wolf populations^32,33^. This population suffered a severe decline since the beginning of the 20^th^ century until the 1970s due to intense persecution^34^. In recent decades, the population has been expanding^32^: most recent estimates put the population at >2,000 individuals in >350 packs, distributed over ca. 140,000 km^2^ ^32,35,36^. A large part of this population occurs in human-dominated landscapes^37–39^ and is remarkable for feeding mainly on anthropogenic sources of food in some areas^40–42^.

Taking advantage of an extensive collection of genotype and collar-tracking data, collected over approximately 20 years, we examined the spatial genetic population structure and spatial behaviour in the Iberian wolf population. We used Bayesian clustering methods to identify geographically and genetically meaningful groups and combined this information with the location of sampled individuals, as well as information about the spatial behaviour of the tracked wolves to investigate the spatial organization of these groups. In particular, we explored (i) the distinct genetic groups that can be identified in the NW Iberian wolf population, (ii) the spatial organization and connectivity among these groups, and (iii) the partition of genetic diversity among groups. This integrated approach revealed a marked fine scale population structure within the Iberian wolf population, with a very low number of disperser individuals among groups and consequently, low levels of admixture. The genetic patterns are confirmed by the spatial behaviour information, providing further evidence for the extremely low dispersal in this wolf population.

## Results

### Genetic clustering analysis and identification of admixed individuals

**Figure 1:**
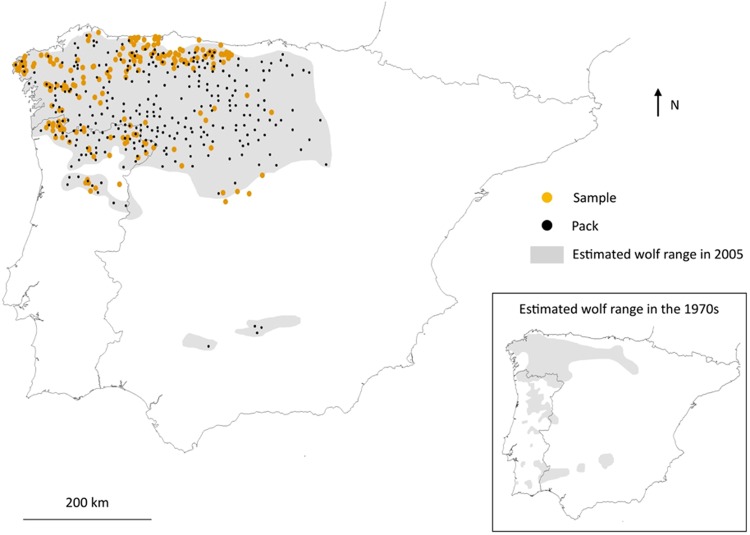
Estimated wolf range in the Iberian Peninsula in 2005 and in the 1970s. Estimated wolf pack locations in 2005 were represented according to^55^, and are denoted with black dots. The locations of the genetic samples used in this study are shown in orange. Historical wolf range is based on^34,60^.

We based our genetic analyses on a dataset of 218 individual wolves genotyped at 46 microsatellite loci, spanning the entire distribution in the Iberian Peninsula (Fig. 1). We explored the genetic structure of the population using the multivariate approach of *DAPC*^43^ and the Bayesian clustering analyses of *Structure*^44^ and *BAPS*^45^.

**Figure 2:**
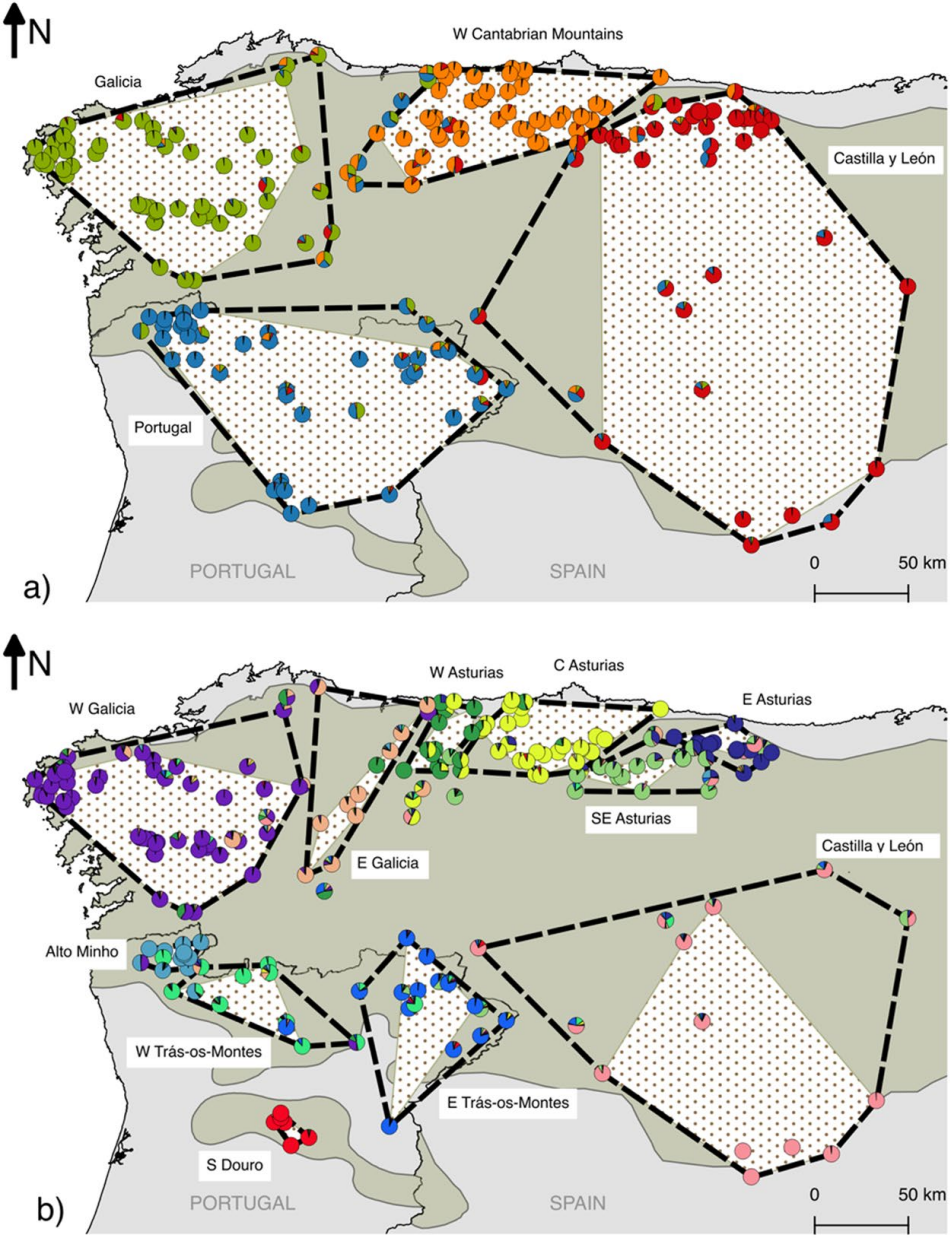
Membership proportions of wolves sampled in this study, according to the *Structure* analysis for K = 4 (**a**) and K = 11 (**b**). Polygons encompassing all individuals of each geographic population (P_total_) and only non-admixed individuals (P_non-admixed_) are represented as dashed black lines and white dotted areas, respectively. The shaded area represents the wolf distribution in the Iberian Peninsula.

Bayesian clustering analyses with *Structure*^44^ showed an increase in posterior probability values Pr(X|K) with increasing values of K (i.e., the number of assumed genetic clusters), while ΔK values^46^ peaked at K = 2 and K = 4 (Supplementary Fig. S1). Results between *Structure* runs for the same K were generally very consistent, with variance increasing for higher values of K (Supplementary Fig. S1). Determining the number of groups from *Structure* runs is not straightforward: the program authors warn that Pr(X|K) serves only as indication of the true number of groups and biological plausibility has to be taken into account^44^ (manual of the program). The ΔK statistic was developed as a further aid to this task, by measuring the rate of change in the probability of data between successive values of K^46^. While K = 2 showed the highest ΔK value, this partitioning seems to reflect a West-East continuum of membership proportions (Supplementary Fig. S2), and log-likelihood values still increased substantially for higher K values (Supplementary Fig. S1). The next highest substantial change in ΔK was observed at K = 4, whereas the highest posterior probability values for which geographically meaningful patterns could still be discerned was K = 11 (Fig. 2; Supplementary Figs S1 and S2). Also, among higher values of K (K > 8), K = 11 was the only value to produce the same clustering pattern in all 20 iterations (Supplementary Fig. S2). For K > 12, the variance between runs increased substantially (Supplementary Fig. S1).

Performing a hierarchical analysis of the clusters starting at K = 2 (i.e. running *Structure* independently for each of the clusters identified at K = 2) results in a similar pattern as K = 4 in the general analysis (Supplementary Fig. S3): within each of the clusters, the best number of K, as determined by the ΔK values, was also two, and the general assignment of individuals was the same. Repeating the same approach for each the four clusters leads to the identification of a total of 13 clusters, eight of which correspond to the same clusters identified previously at K = 11 in the general analysis (Fig. 2; Supplementary Fig. S3). The remaining five clusters result from a division of one cluster at K = 11 into four, and the merging of two others (Supplementary Fig. S3).

**Figure 3:**
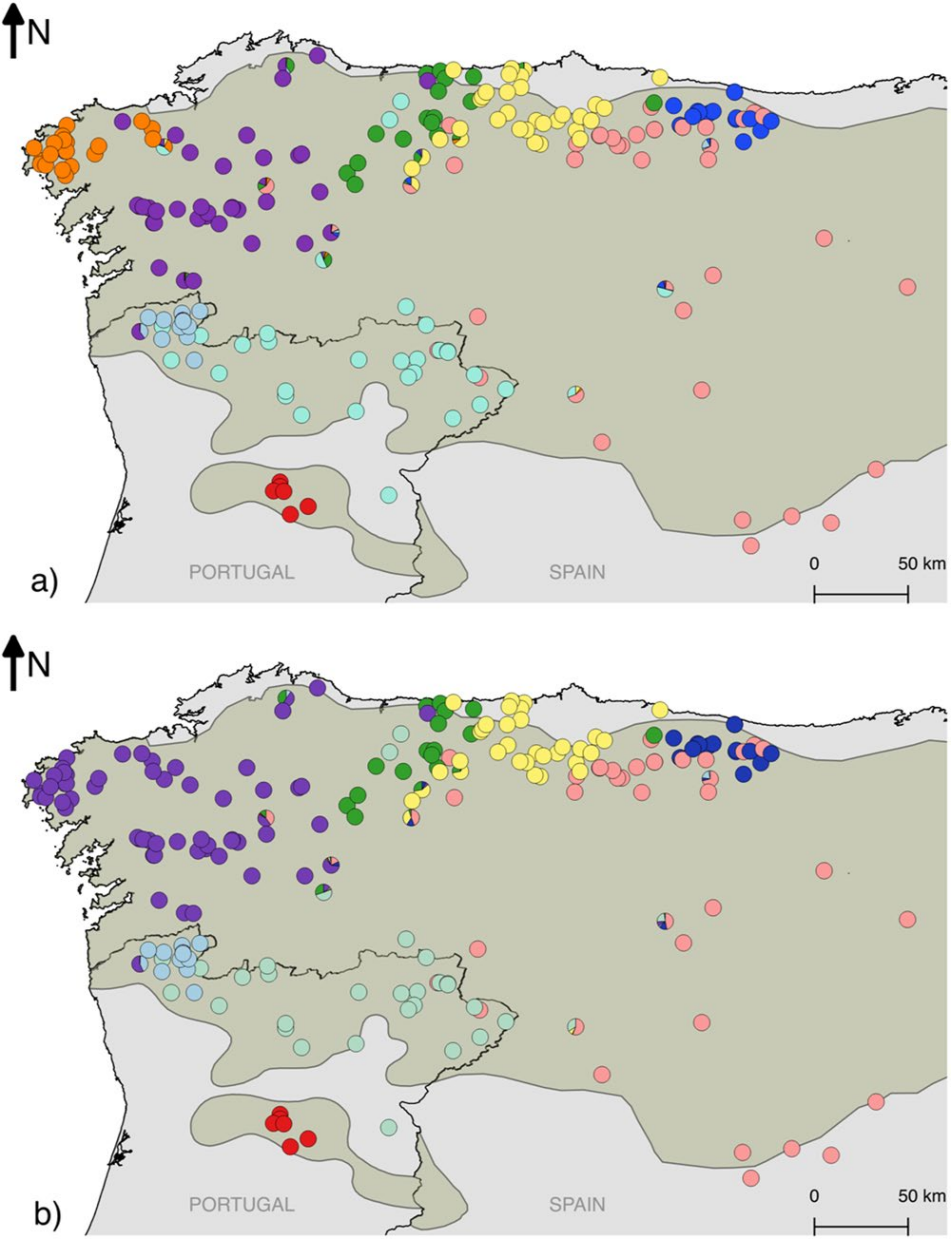
Membership proportions of wolves sampled in this study, according to *BAPS*, using the regular (non-spatial) (**a**) and spatial (**b**) admixture analyses. The shaded area represents the wolf distribution in the Iberian Peninsula.

Clustering analyses with *BAPS* identified K = 9 as the best partition in regular mode (without spatial information) and K = 8 in the spatial clustering. Compared to the *Structure* analysis at K = 11, some clusters merge but maintain their general pattern (Fig. 3). Bayesian Information Criterion (BIC) values in *DAPC* steadily decreased with increasing number of clusters, with minimum values between eight and eleven, before rising again for higher values (Supplementary Fig. S4). To explore the population structure with maximum detail, and make the results comparable with those from the *Structure* analysis, we discuss the *DAPC* results at eleven clusters.

Given the results from the different clustering methods, and the lack of any clear rules in determining the exact number of clusters, we chose to prioritize the geographical sense of the data in this study. K = 2 appears to represent a longitudinal gradient that results from isolation by distance (Mantel test p = 0, one-tailed), and not from population clustering (Supplementary Fig. S2); while this may be indicative of reduced dispersal of individuals in the dataset, K = 2 does not therefore constitute a meaningful clustering solution. Furthermore, it has also been described that the application of the ΔK statistic tends to pick K = 2 as the best number of clusters even when further substructure is present^47^. Given the second increase in ΔK at K = 4 (Supplementary Fig. S1), and that K = 11 appears as the maximum number of clusters for which a geographical patterns can still be discerned (Supplementary Fig. S2), we consider these two levels of genetic structure for the subsequent analyses. The results from both *BAPS* analyses and *DAPC* confirm most of the clusters identified with *Structure*, although at less resolution. We consider that both K = 4 and K = 11 capture the genetic structure of the Iberian wolf population, albeit at different levels.

Based on the partition in K = 4 and K = 11, we classified individuals into genetically ‘non-admixed’ and ‘admixed’ based on their membership proportions to the inferred clusters by *Structure* (see Methods). Average cut-off values to differentiate between ‘non-admixed’ and ‘admixed’ individuals were very high, both for K = 4: 0.83 (0.77–0.87), and for K = 11: 0.88 (0.81–0.95) (Supplementary Figs S5 and S6). ‘Non-admixed’ individuals exhibited very high membership proportions (*q*_*i*_), K = 4: 0.94–0.96, K = 11: 0.90–0.97. In total, 166 (76%) and 137 (63%) out of 218 individuals were classified as ‘non-admixed’, for K = 4 and K = 11, respectively (Tables 1 and 2).

**Table 1:** Statistics for geographical genetic groups of Iberian wolf at K = 4.

	Genetic group			
	W Cantabrian Mountains	N Portugal	Castilla y León	Galicia
Number of individuals	52	47	55	59
**Mean membership proportions to genetic group**				
W Cantabrian Mountains	0.89	0.04	0.07	0.03
N Portugal	0.04	0.85	0.08	0.03
Castilla y León	0.06	0.04	0.83	0.03
Galicia	0.02	0.07	0.02	0.91
Number of genetic groups contributing >5%	1	1	2	0
Number of admixed individuals (% of total)	6 (12%)	12 (28%)	19 (35%)	10 (17%)
Number of dispersers from other genetic groups (%)	0 (0%)	0 (0%)	0 (0%)	0 (0%)

**Table 2:** Statistics for geographical genetic groups of Iberian wolf at K = 11.

	Alto Minho	E Trás-os-Montes	SE Asturias	W Asturias	Castilla y León	S Douro	W Galicia	C Asturias	E Galicia	W Trás-os-Montes	E Asturias
Number of individuals	13	17	21	12	14	6	53	30	11	11	19
**Mean membership proportion to genetic groups**											
Alto Minho	0.82	0.01	0.00	0.00	0.00	0.03	0.01	0.00	0.02	0.08	0.00
E Trás-os-Montes	0.02	0.73	0.01	0.01	0.04	0.01	0.01	0.01	0.02	0.11	0.02
SE Asturias	0.01	0.08	0.77	0.08	0.06	0.00	0.01	0.01	0.01	0.01	0.10
W Asturias	0.00	0.01	0.01	0.77	0.01	0.00	0.02	0.07	0.10	0.02	0.01
Castilla y León	0.01	0.01	0.02	0.00	0.78	0.01	0.01	0.01	0.01	0.01	0.07
S Douro	0.00	0.02	0.00	0.00	0.01	0.91	0.00	0.01	0.00	0.01	0.00
W Galicia	0.04	0.01	0.00	0.00	0.01	0.01	0.84	0.00	0.05	0.04	0.00
C Asturias	0.00	0.01	0.03	0.09	0.01	0.00	0.01	0.85	0.01	0.02	0.02
E Galicia	0.01	0.02	0.01	0.01	0.01	0.01	0.06	0.01	0.76	0.05	0.02
W Trás-os-Montes	0.08	0.09	0.02	0.01	0.04	0.01	0.01	0.00	0.01	0.66	0.01
E Asturias	0.00	0.01	0.12	0.02	0.03	0.00	0.00	0.02	0.01	0.00	0.76
**Number of genetic groups**											
contributing >5%	1	2	1	2	1	0	1	1	2	2	2
Number of admixed individuals (% of total)	2 (15%)	10 (59%)	8 (38%)	4 (33%)	5 (36%)	1 (17%)	17 (32%)	9 (30%)	4 (36%)	5 (45%)	7 (37%)
Number of dispersers from other genetic groups (%)	1 (8%)	0 (0%)	0 (0%)	1 (8%)	0 (0%)	0 (0%)	0 (0%)	0 (0%)	1 (9%)	1 (9%)	1 (5%)

### Spatial configuration of genetic clusters

**Figure 4:**
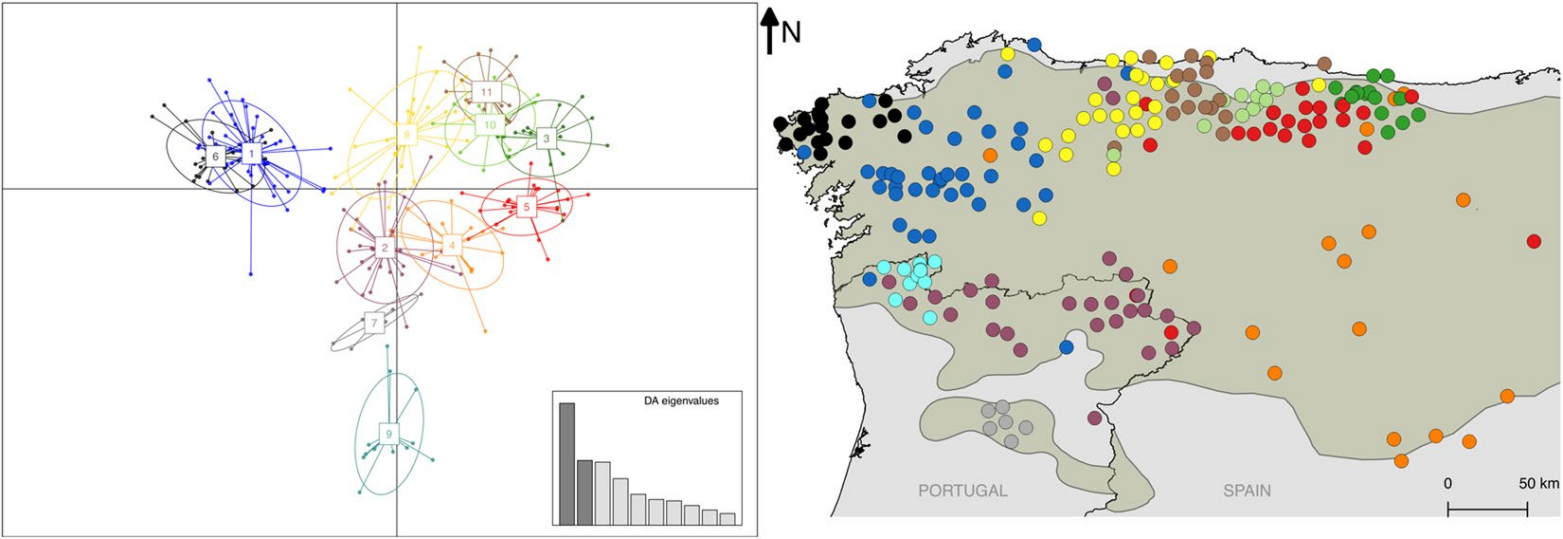
Results of the clustering analysis in *DAPC* assuming eleven clusters. The placement of individual samples along the first two principal components axes is represented in the scatter plot (left), where each of the identified clusters is represented in a different colour. The map (right) represents the inferred assignment of each individual to the clusters, using the same colours.

The spatial projection of wolf samples revealed that for K = 4 the identified genetic clusters in the *Structure* analysis corresponded to the regions of Galicia (NW Spain), N Portugal, the W Cantabrian Mountains (N Spain), and a large area extending from the E Cantabrian Mountains to the plateau of Castilla y León (Fig. 2a). At K = 11 we identified four groups in Portugal (Alto Minho, W Trás-os-Montes, E Trás-os-Montes and S Douro, the latter being the only one entirely located to the south of the Douro river) and seven groups in Spain (W Galicia, E Galicia, W Asturias, C Asturias, E Asturias, SE Asturias and Castilla y León) (Fig. 2b). The E and SE Asturias groups showed greater affinity with the group of Castilla y León, since individuals from these groups cluster together at K = 4. Similarly, the cluster of Castilla y León is joined with SE Asturias in the hierarchical *Structure* analysis (Supplementary Fig. S3) and both *BAPS* analyses (Fig. 3). The group designated E Galicia at K = 11 included individuals from both the Galician and Cantabrian groups at K = 4. Smaller clusters are identified within the large W Galician cluster by running *Structure* in a hierarchical manner (Supplementary Fig. S3), and in the regular BAPS clustering analysis (Fig. 3). Grouping individuals in eleven clusters using *DAPC* joins the two clusters of Trás-os-Montes (W and E) and divides the W Galician in two (Fig. 4).

**Figure 5:**
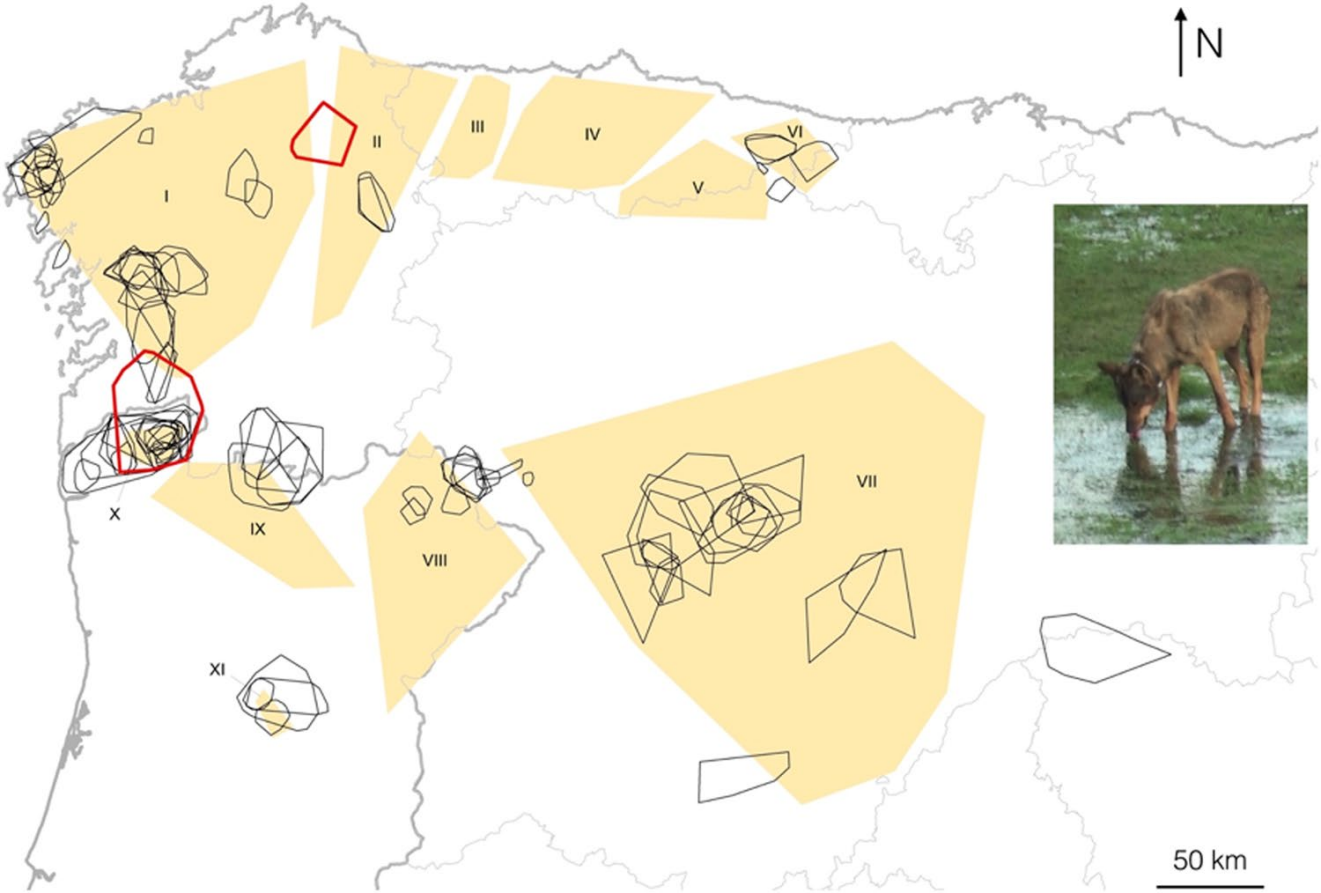
Spatial overlap between the genetic groups identified at K = 11 (orange polygons) and the full monitoring MCPs (100% of locations, hollow polygons) for 85 wolves collared in the Iberian Peninsula between 1982 and 2015. Wolf MCPs in red represent the only two cases observed with a MCP overlapping more than one genetic group. See Supplementary Table S3 for details. Roman numbers in the orange polygons denote genetic groups. I: W Galicia, II: E Galicia, III: W Asturias, IV: C Asturias, V: SE Asturias, VI: E Asturias, VII: Castilla-León, VIII: E Trás-os-Montes, IX: W Trás-os-Montes, X: Alto Minho, XI: S Douro. Wolf photograph courtesy of Francisco J. Lema.

At K = 4, the identified geographical groups contained a similar sample size (range 47–59 individuals) (Table 1). However, a higher variability was observed in sample sizes at K = 11. The S Douro group showed the smallest sample size (6 individuals), while W Galicia was the group more intensively sampled (53 individuals) (Table 2). Individual membership proportions to genetic clusters within their respective geographic groups were, on average, higher for K = 4 (mean Q_i_ over populations: 0.87) than for K = 11 (mean Q_i_ over populations: 0.79). Surprisingly, in both cases the genetic contribution from external groups was extremely low at both levels. Each group received the genetic contribution of only one or two other groups (5% threshold), indicating low exchange of genes among groups, and not always from the closest ones (Tables 1 and 2). Groups from E and SE Asturias stand out as presenting substantial mutual genetic contribution (>0.10), while on the other hand, the isolation of the group of S Douro was remarkable. Admixed individuals represented on average 23% of each group for K = 4 (range 12–35%), and 34% for K = 11 (range 15–59%) (Tables 1 and 2).

Overall, for each genetic group, most individuals within groups (89–94%) occurred within the area defined by the most external ‘genetically non-admixed’ individuals for each group (P_non-admixed_) (Tables 1 and 2; Fig. 2). 64% and 49% of admixed individuals were found inside the P_non-admixed_ of their genetic group, for K = 4 and K = 11, respectively (Tables 1 and 2). P_non-admixed_ areas represented on average 84% (74–92%) and 63% (22–100%) of P_total_ areas at K = 4 and K = 11, respectively (Tables 1 and 2).

The proportion of individuals classified as dispersers in our genetic dataset (i.e. those individuals showing a membership proportion to one genetic cluster above the cut-off value but found within the P_total_ of another group) was extremely low. No dispersers were integrated in any of the groups at K = 4 (Table 1), although two female individuals were identified as dispersers originally from the N Portugal and Galician groups. However, we were not able to geographically attribute any of these females to a given genetic group since their location did not overlap with any group. At K = 11 level, at most one disperser per group was found, and only five out of the eleven groups had such a disperser. In total, we identified seven dispersers (two females, five males) at K = 11, representing 3.2% of our total dataset (Table 2). With the spatial resolution obtained from our dataset, we were not able to assign two out of seven dispersers to any of the genetic groups identified. Dispersers originated from E Trás-os-Montes, W Trás-os-Montes, W Asturias, C Asturias, SE Asturias, and W Galicia.

*BayesAss* identified the above mentioned five dispersers as first-generation migrants, and confirmed their populations of origin. Additionally, three other individuals were classified as first-generation migrants with probabilities >90%. One individual in E Asturias, coming from SE Asturias, and one in E Galicia, coming from W Galicia, were classified as ‘admixed’ based on their admixture proportions in *Structure*. The third individual from E Galicia was previously classified as ‘non-admixed’.

Information from collared wolves supported the patterns observed using genetic analyses. First, we found a remarkable geographical overlap between genetic groups and wolves’ P_s_, defined as the minimum convex polygon using all recorded spatial locations during every wolf study period (i.e. monitoring time) (Fig. 5; Supplementary Table S3). Second, the majority (97.6%, n = 85) of wolves’ P_s_ did not overlap with more than one P_total_ defined from our genetic dataset. Only 2 out of 85 collared wolves (2.3%) overlapped with more than one genetic group (Fig. 5).

### Genetic diversity and differentiation

Due to the low number of dispersers found in our dataset, genetic diversity values did not change substantially when considering either geographical groups or genetic clusters, both at K = 4 and K = 11 (Supplementary Tables S4 and S5). For K = 4, genetic diversity was similar between all groups, as measured by the mean expected heterozygosity (H_e_), varying between 0.57 and 0.62 (mean H_e_ = 0.59) (Supplementary Table S4). At K = 11, H_e_ values were more variable between groups, ranging between 0.48 and 0.59 for geographical groups (mean H_e_ = 0.55) (Supplementary Table S5). The lowest H_e_ values were observed in E Asturias and S Douro groups (H_e_ = 0.48 in both cases), the latter also presenting the lowest allelic richness. Furthermore, the S Douro group showed a higher private allelic richness (pAR = 0.15) compared to the other groups at K = 11 (pAR ≤ 0.11). On the contrary, E Trás-os-Montes and E Galicia showed the highest H_e_ and allelic richness values.

We did not detect signs of inbreeding across groups, as observed and expected heterozygosities were similar, leading to F values close to zero (Supplementary Tables S4 and S5). At K = 4, we did not detect close relationships among groups, as relatedness values were always <0.2 (Supplementary Table S4). However, mean relatedness values were slightly higher at K = 11, with three groups presenting mean relatedness values that would be expected for half-siblings (i.e., r ≥ 0.25): S Douro (r = 0.38), E Asturias (r = 0.25–0.32) and Alto Minho (r = 0.25–0.28) (Supplementary Table S5).

At K = 4, all groups appeared similarly differentiated, as measured by pairwise F_ST_ (0.06–0.11) (Supplementary Table S6). For K = 11, pairwise F_ST_ values were more variable (0.03–0.25), the highest differentiation being observed between S Douro and E Asturias, and the lowest between E Trás-os-Montes and W Trás-os-Montes (Supplementary Table S7). On average, the S Douro group showed the highest pairwise F_ST_ differentiation with all other groups despite its close geographic proximity (average F_ST_: 0.19;), while the neighbouring E Trás-os-Montes and W Trás-os-Montes showed the lowest differentiation (average F_ST_: 0.09). Results implementing Jost’s D distance were similar (Supplementary Tables S8 and S9).

Migration rates between clusters inferred by *BayesAss* were very low, with most 95% credible values including zero. Migration was only found to be significant (i.e. inferred migration rates whose 95% credible values did not include zero) between the two Galician clusters and between the clusters in Asturias (Table 3).

**Table 3:** Migration rates (mean ± 95% credible intervals) between Iberian wolf genetic subgroups inferred by BayesAss. Only values that do not overlap zero are presented.

Subpopulations involved	Migration rate
W Asturias -> C Asturias	0.0411 ± 0.036
SE Asturias -> E Asturias	0.0629 ± 0.048
C Asturias -> W Asturias	0.0531 ± 0.047
W Galicia -> E Galicia	0.1542 ± 0.073

## Discussion

In this study, we investigated the genetic structure of the Iberian wolf population and the patterns of gene flow across identified genetic clusters. Additionally, we used spatial information from collared wolves to understand effective dispersal levels across genetic groups. Given the recent history of fragmentation and decline of this population^32,34^, we expected possible signals of population structure to be reflected on fast-evolving molecular markers such as microsatellites and therefore genotyped a comprehensive sample of 218 individuals at 46 such loci.

We identified four main genetic groups in the NW Iberian wolf population (Fig. 2a). At this level, there are no apparent large-scale topographical barriers nor gaps in the wolf range that would provide a straightforward explanation for this partition (Supplementary Fig. S7), revealing the need for further studies that explicitly take environmental features into account. A more detailed analysis of the genetic structure of this population led to the identification of up to 11 geographically coherent genetic groups, a remarkable number considering the relatively small wolf range of this population (ca. 140,000 km^2^) and the generalist behaviour and mobility capabilities of wolves^22^. The size and genetic diversity of these fine-scale groups was very variable: four distinct genetic groups were consistently identified along the Cantabrian mountain range in all our analyses; two to three groups appear to be present in Portugal, north of the Douro river; and most of the regions of Galicia and Castilla y León seem to be occupied by wolves from a single genetic group, although some smaller groupings could be identified in W Galicia. It should be noted that our sampling is sparser in the central and eastern parts of the Iberian wolf distribution (NW and E of the Spanish region of Castilla y León, respectively), where wolf pack density is also lower (Fig. 1). This makes it difficult to establish with more precision the limits of the surrounding clusters, which probably extend to different degrees into the less well-sampled areas.

Our results are consistent with other studies showing that wolf populations can be characterized by relatively low levels of gene flow and short dispersal distances, despite the potentially high dispersal capabilities of the species^26,28,29^. However, in the case of the NW Iberian wolf population, our results show a remarkably reticulated genetic population structure at such a fine scale that is not comparable with previous reports from other wolf populations. Genetically differentiated wolf subpopulations have been described at continental scales, where habitats are more heterogeneous and gaps in population ranges exist, allowing for substantial genetic drift and adaptation to specific ecological conditions^3,48–50^. However, at smaller spatial scales the number of described subpopulations or genetic groups is usually lower^10,49,51^.

The identified genetic groups appear to be characterized by very low levels of admixture and moderate to high genetic differentiation (F_ST_). The number of wolves identified as dispersers was extremely low, both in the genetic dataset (3.2–3.7%, n = 218) and in the collared wolves dataset (2.3%, n = 85). Furthermore, genetic and behaviour information were extremely consistent in the geographical configuration of genetic groups: information about the spatial behaviour of wolves was available for 9 of the 11 genetic groups, and in all of them except two individual cases, wolves spent the entire monitoring time within the area defined by the genetic samples (P_total_) (Fig. 5). Our results therefore suggest that effective dispersal outside the genetic group where individuals are born is a rare phenomenon in the NW Iberian wolf population. This is in accordance with a previous study based on nine collared wolves in Castilla y León that found a mean natal dispersal distance of 32 km^37^.

Several environmental conditions have been found to constrain the dispersal of wolves and other mobile carnivores, including habitat-specific adaptations to prey or climate^52,53^, population density^23–25^ or anthropogenic pressure^10,15^. Nevertheless, prior evidence on wolf range and feeding ecology in the Iberian Peninsula^38,40^ does not seem to explain the genetic discontinuities identified in this study, suggesting these may not be the main drivers of wolf genetic structure in this region, although possible patterns of habitat or prey selection deserve further investigation. Our extensive sampling will allow for future testing of the association between genome-wide markers and environmental factors to test the hypothesis of the existence of local adaptive genetic variation. Geographically, some overlap appears to exist between the boundaries of some of the eleven identified genetic groups and main rivers, namely in Northern Portugal, Galicia and Western Asturias (Supplementary Fig. S8), but the influence of these features requires further study. Spatially-explicit landscape genetic approaches will be applied in the future to ascertain the degree to which major natural or anthropogenic features, such as rivers, mountains or highways, may influence the observed gene flow patterns and spatial genetic structure. Regarding population density, it has been described that the tendency for wolves to disperse larger distances into new territory seems to be more common in situations of high density and intense resource competition^25^. Wolf density in some regions of the Iberian Peninsula are among the highest in Europe (up to 7 individuals/100 km^2^)^54,55^; on the other hand, Iberian wolves are also remarkable for their reliance on livestock, which is relatively abundant and available^38,40,41^, possibly explaining the maintenance of high wolf population densities with reduced dispersal.

Other factors deserving further study are those resulting from anthropogenic pressure: infrastructure development, heterogeneous human persecution of wolves and management strategies, and other socio-economic trends can cause local extinctions and induce fragmentation or promote source-sink dynamics^56,57^. For example, wolf protection and population management practices in the Iberian Peninsula vary greatly depending on regional jurisdictions, leading to a heterogeneous human pressure: wolves north of the Douro river in Spain are listed in Annex V of the European Habitats Directive (92/43/EEC), being either game species with hunting quotas, game species without hunting quotas, or subject to a special regime, depending on the Spanish autonomous region; wolves south of the Douro river in Spain and in the entire Portuguese territory are fully protected, being listed in Annexes II and IV of the EU Habitats Directive. It is also worth mentioning that the genetic group of Castilla y León encompasses areas under both Annexes of the directive, with wolves being listed either as game species or being fully protected. Nonetheless, illegal persecution is also common throughout the entire wolf range, both in Spain and Portugal^37,58^.

The Iberian wolf population has been affected by centuries of human persecution and anthropogenic habitat changes, which led to periods of severe decline and fragmentation. Unlike the rest of Europe, where wolf populations are showing positive trends^32^, at least some parts of the Iberian wolf population are still declining, particularly the populations of Sierra Morena^59^ and South of the Douro river in Portugal^60^. While at K = 4 the identified groups showed similar levels of genetic diversity, a larger variability was found at K = 11, which might reflect distinct local population trajectories. Larger and more diverse groups might represent areas where wolf abundance has been consistently high even during the population minimum in the 1970s, such as the genetic groups detected in Galicia or the Cantabrian mountains^34,61,62^ (see Fig. 1 for the estimated wolf range in the Iberian Peninsula in the 1970s). The two different scales of our analysis also reveals that wolves occupying the plateau of Castilla-León, reaching the Madrid and Guadalajara provinces in recent times, result from the expansion of wolves from the SE Cantabrian Mountains (i.e., it merges with the E and SE Asturias populations at K = 4), which has been described to have served as a refuge for this population during the minimum peak population level at the 1970s^34^. The genetic identity of some of the smaller groups identified, like S Douro or Alto Minho, might be explained by the isolation imposed from this fragmentation after the 1970s, which then might have been reinforced over time by the low dispersal discussed above.

Analysing in detail the genetic contribution of each of the eleven genetic groups to each other in a geographical context, there appear to be two main routes for gene flow: in the North, along the range of the Cantabrian Mountains, and to the South, along the regions North of the Douro river in Portugal. The isolation of the S Douro group in Portugal is also evident from these results, despite its close geographic proximity to other wolf populations (ca. 20 km). Over the past century, wolf populations North of the Douro river have maintained a larger degree of connectivity, while the populations South of this river became very fragmented, with an estimated maximum number of 16 population fragments in the 1960–70s^60^. Currently, the S Douro population in Portugal represents the sole survivor of these fragments, estimated to be comprised of 6 packs with a population size of ca. 30 individuals^63^. This population has a low reproductive success, having declined by 58% from 1990 to 2002, its largest range decline since the beginning of the 20^th^ century^60^.

The genetic structure of a population results from a complex interaction between ecological traits, environmental features affecting gene flow and historical events. Given the lack of an extensive sampling before the population decline and fragmentation in mid-20^th^ century, it is difficult to assess from extant genetic data how much of the observed genetic population structure results from the anthropogenic impacts of the last decades and the recovery of wolves in recent decades, or if it reflects a more long-term trait of the Iberian wolf population, determined by ecological or geographical features. Furthermore, information on how population genetic structures last over large time periods is still scarce^60^. Also, given that patterns of isolation by distance and genetic clustering are difficult to distinguish^64,65^, further work is needed to clarify the relative influence of these patterns on the structure of the Iberian wolf population.

The results of our work are also relevant for the management and conservation of the Iberian wolf population. The Wolf Specialist Group of the Species Survival Commission of the International Union for the Conservation of Nature (IUCN) recognized in point 3 of its Wolf Manifesto ‘that wolf populations have differentiated into entities which are genetically adapted to particular environments’ and that ‘it is of first importance that these local populations be maintained in viable populations in their natural environments in a wild state’. The Iberian wolf population is the largest remaining population in Western Europe, but is also currently isolated, potentially representing a unique population from the genetic, behavioural and ecological perspectives. Furthermore, genetic diversity is one of the three categories of biodiversity for conservation priority recommended by the IUCN^66^. Identifying conservation units below the population level^67^, particularly in relation to CPS, their connectivity and long-term viability are increasingly recognised as important goals for conservation (because of their potential evolutionary adaptive value) and population management (because the same population is often heterogeneously managed or because different populations are managed as a single unit). However, few attempts have been made to integrate this type of information into management^68^. Our results bring the opportunity to open a debate of this topic and integrate information regarding CPS into the NW Iberian wolf population management.

## Methods

### Sample collection and selection

Between 1995 and 2014, we collected 289 wolf tissue samples in Portugal and Spain (Fig. 1) from dead animals (mainly road kills, poached, legally culled or hunted wolves). Animals were never specifically killed for this study. For each tissue sample we recorded the geographic coordinates (latitude and longitude) where the sample was collected, the sex and, when possible, the approximate age of the animal. Age was estimated by dental pattern and wear, according to Gipson *et al*.^69^, and physical development. Our sampling encompasses ca. 80% of the wolf estimated range in 2005 in the Iberian Peninsula^35,55,63,70^.

Because several sampled individuals could belong to the same pack, we used the following criteria to avoid close familiar relationships in our dataset: (i) we plotted all samples against the wolf packs detected between 1993 and 2005 (Fig. 1) and calculated the Euclidean distance among all tissue sample locations; (ii) for samples separated by less than 10 km, and/or within the same buffer area (100 km^2^) of wolf packs (centred on the estimated breeding sites in the correspondent period), we noted the date in which every sample was collected; (iii) considering the annual cycle of wolves (from births in May of a given year to the next May), we focused on those samples separated ≥5 yrs. This criteria ensured that more than 1 generation spanned between samples (mean generation time in wolves is estimated to be 3–4 yrs^71,72^); (iv) we selected a maximum of two samples for each 5-yr temporal window. To do this, we took into account preliminary information of missing data from microsatellite analyses (selecting those samples with the best performance), and the age of individuals, prioritizing adult individuals (>2 yrs.). Samples with >20% missing data were also excluded for subsequent analyses. Given the occurrence of wolf-dog hybridization in the Iberian Peninsula^73^, possible hybrids were eliminated based on genetic data, using the procedure and dog reference samples in Godinho *et al*.^73^. The final genetic dataset used for this study contained 218 individuals.

### DNA extraction, markers and genotyping

Total genomic DNA was extracted using the QIAGEN DNeasy Blood & Tissue Kit (Qiagen, Valencia, CA, USA) according to the manufacturer’s instructions. DNA quality and concentration were assessed using agarose gel electrophoresis and quantified in a Qubit fluorometer (Thermo Fisher Scientific, Wilmington, DE, USA). Samples were amplified for a set of 46 microsatellite loci in four multiplex reactions (MS1 to MS3, and the Canine Genotypes Panel 2.1 Kit, Thermo Fisher Scientific), and a singleplex including the Dbar2 locus, following Godinho *et al*.^73,74^ (Supplementary Tables S1 and S2). PCR products were run on an ABI3130xl Genetic Analyser (Applied Biosystems, Waltham, Massachusetts, USA) using GeneScan500 LIZ size standard. The results were checked manually, and alleles scored, using *Genemapper* 4.1 (Applied Biosystems).

### Clustering analysis

We employed the Bayesian clustering algorithms of *Structure* 2.3.3^44^ and *BAPS* 6^45^ to group all samples into clusters using their multilocus genotypes.

*Structure* was run for 2 million iterations after 500 thousand burn-in steps, using the admixture and correlated allele frequencies models, assuming values of the K parameter (number of populations) between 1 and 20. For each K, runs were repeated 20 times to ensure the consistency of the results. To select the most likely number of groups we tabulated the posterior probabilities of the data for each K (ln Pr(X|K)) and employed the procedure proposed by Evanno *et al*.^46^, which involves calculating the quantity ΔK for each pair of successive Ks, using *StructureHarvester*^75^. Having chosen K, we classified each individual in each cluster *i* based on the distribution of the individual membership proportions to that cluster (*q*_*i*_). A cut-off value was set for each cluster at the point where a gap in the distribution of membership proportions was visible, and above which membership proportions no longer varied considerably between individuals (Supplementary Figs S5 and S6). Individuals above this threshold were considered to fully belong to the respective cluster (i.e., ‘non-admixed’ individuals), while all individuals below the cut-off point were considered ‘admixed’, i.e., having a genetic background in more than one genetic cluster (Supplementary Figs S5 and S6). To confirm the clustering results, the same *Structure* analysis was run in a hierarchical manner, progressively dividing the samples according to the clusters they were placed in. In the first iteration of this procedure, *Structure* was run separately on each of the two main clusters identified (see Results) for K between 1 and 5, and used to calculate ΔK values; in the second iteration, samples were again divided according to the inferred clusters and the procedure was repeated.

Clustering of individuals was also performed in *BAPS* with both the regular (non-spatial) and spatial population mixture analysis, starting from different values of the maximum number of clusters (K), up to K = 20. For each K, 20 replicate runs were performed. Individual admixture proportions were then calculated based on each of the clustering analyses using the ‘admixture of individuals based on mixture clustering’ option.

Additionally, the multivariate method *DAPC*^43^ was used to identify clusters of genetically related individuals, as implemented in the R package adegenet 2.0^76,77^. *DAPC* is a model-free method, and therefore free of population genetics assumptions, in particular Hardy-Weinberg equilibrium. The function ‘find.clusters’ was used with 80 principal components (PCs, explaining >90% of the variance) to determine the number of clusters maximizing the variation between clusters. The Bayesian Information Criterion (BIC) was used to identify the optimal number of clusters. The number of retained PCs was calculated using a cross validation methods implemented in the ‘xvalDapc’ function.

### Spatial genetic analyses

Samples were spatially projected using QGIS 2.8.2^78^. We grouped samples according to similar posterior assignment probabilities to the different groups identified in our dataset in order to spatially identify geographical clusters. For a given genetic cluster, all adjacent individuals with a membership proportion to the same cluster higher than 0.50 were grouped. These individuals were used to define a minimum convex polygon for each genetic cluster (P_total_). Individuals for which position and/or genetic makeup did not allow for a clear attribution were not included. In a second step, we defined another minimum convex polygon encompassing the area comprising only genetically ‘non-admixed’ individuals (P_non-admixed_). All ‘non-admixed’ individuals that were not located within the P_total_ area of their respective population were considered as dispersers.

For each identified geographical group, we calculated the area of the P_total_ and P_non-admixed_, computed the mean membership proportions, the percentage of ‘admixed’ individuals at the P_total_ level, and within the P_non-admixed_, the number of immigrants (i.e. dispersers from other populations falling with P_total_). Moreover, in order to gain insights into the connection between genetic clusters, we also calculated the number of contributing genetic clusters to the ancestry of each group, by considering only groups with >5% membership proportions, and the smallest distance to the closest group (km, measured as the smallest Euclidean linear distance between P_total_ borders).

We tested for correlation between genetic and geographical distance (isolation by distance – IBD) using all samples in the dataset with a Mantel test implemented in *Genepop* 4.3^79^. Given that Mantel tests and tests of spatial autocorrelation are not capable of distinguishing between patterns resulting from genetic clustering and IBD^64^, we did not attempt to perform further IBD tests at smaller scales.

### Population genetics analyses

For each geographical group, as well as for each genetic cluster identified by *Structure* (including only ‘non-admixed’ individuals), we calculated standard population genetics parameters including: (i) the number of alleles, (ii) observed and expected heterozygosities, and (iii) the fixation coefficient (F), using *GenAlEx* 6.501^80^; (iv) allelic richness and private allelic richness, rarefied to the smallest sample size, using *ADZE* 1.0^81^. We used *Genepop* 4.3^79^ to test for significant deviation from Hardy-Weinberg equilibrium and association between genotypes at pairs of loci (linkage disequilibrium). Statistical significance levels were adjusted using Bonferroni corrections^82^. Additionally, we also estimated the average relatedness between pairs of individuals for each group/cluster using the estimator of Lynch & Ritland^83^ with *GenAlEx*. Differentiation between groups/clusters was measured by F_ST_ (ϕ_ST_) calculated from an AMOVA^84^ (equivalent to Weir & Cockerham^85^), using 1,000 permutations to test their significance, and Jost’s D distance^86^, both calculated using *GenoDive* 2.07b27^87^. A total of three and eight individuals were excluded at K = 4 and K = 11, respectively, for these analyses because they could not be attributed to a particular geographical group.

Additionally we estimated the migration rates (mean number of migrants per generation, Nm) between all pairs of subpopulations and identified migrants using *BayesAss* 3.0.4^88^. The program was run for 20 million MCMC iterations, with a burn-in of 2 million. The allele frequencies, inbreeding coefficients and migration rates parameters were set to 0.3, 0.7 and 0.6, respectively, to adjust acceptance rates. MCMC mixing was assessed visually by observing the trace file with *Tracer*^89^. Consistency of the results was confirmed by comparing 5 runs with different random number seeds.

### Spatial behaviour of wolves

To investigate whether the identified genetic structure was reflected in the spatial behaviour of wolves, we analysed tracking information of 85 collared individuals. This information was collected from 1982 to 2015 in the context of several research projects on the ecology of this species in Portugal and Spain^37,90–98^ (Supplementary Table S3). Apart from information collected from previously published research (see references^37,90–98^ for respective capture and handling details), all experimental procedures that included the capture, handling and collaring of wolves were specifically approved by the national authorities in Portugal (Instituto da Conservação da Natureza e das Florestas – ICNF) under permits 326/2007/CAPT, 259/2008/CAPT, 261/2009/CAPT, 330/2010/CAPT, 332/2010/MANU, 20/2012/CAPT, 25/2012/MANU, 26/2012/MANU, 27/2012/MANU, 371/2012/CAPT, 229/2015/CAPT, 230/2015/CAPT; and Spain (Regional Government of Galicia and Organismo Autónomo de Parques Nacionales), with permits 19P72009, 481/2011, 1389/2013, CO709/0164/2013, 526/2015, 19/2006, 71/2009 and 86/2011. All field procedures and animal handling were carried out in accordance with animal welfare regulations of Portugal and Spain and followed the guidelines of the American Society of Mammalogists^99^.

A total of 85 wolves were equipped with VHF collars (Televilt, Sweden and Telonics, USA; N = 30) or GPS-GSM/Iridium collars (Followit, Sweden and Vectronic, Germany; N = 55). Our dataset was composed by 39 females and 46 males and contained information from 41 wolves with an estimated age <2 years and 44 wolves with an estimated age ≥2 years. Monitoring period (defined as the time elapsed between the first and last location obtained for each wolf) averaged 378 days (overall range: 16–2,129 days; VHF collars: mean = 678 days, range = 44–2,129; GPS collars: mean = 214 days, range = 16–632); and the mean number of locations per wolf was 2685 (overall range 15–13,709; VHF collars: mean = 163, range = 15–417; GPS collars: mean = 3,573, range = 153–13,709) (Supplementary Table S3).

For each wolf, we calculated a minimum convex polygon (P_s_, equivalent to the MCP 100%) using all of the VHF/GPS locations during the entire monitoring time. Since in this study we were interested in detecting overlaps between individual wolf P_s_ and the spatial distribution of the detected genetic groups (P_total_), we maximized potential overlaps by considering the entire monitoring period of wolves regardless of changes in social status over time. Overall, the mean full monitoring P_s_ for wolves was 408 km^2^ (range 14–2,810) (Supplementary Table S3). Subsequently, we counted the number of genetic groups (i.e., the number of P_total_) overlapping with every individual wolf P_s_ in space.

## Electronic supplementary material

Supporting Information

## Data Availability

Microsatellite genotype data and genetic cluster assignment information was uploaded to the Open Science Framework repository under the identifier 10.17605/OSF.IO/NVSQT.
