# Supplementary material for: Cryptic population structure reveals low dispersal in Iberian wolves
Source: Sci Rep. 2018 Sep 20;8:14108. doi: 10.1038/s41598-018-32369-3 (PMC6147861; doi:10.1038/s41598-018-32369-3)
Supplement: Supplementary file 1 — Supporting Information [file 41598_2018_32369_MOESM1_ESM.pdf]

Supporting Information for

Cryptic population structure reveals low dispersal in the Iberian wolf

Pedro Silva[1,2], José Vicente López-Bao[3], Luis Llaneza[4], Francisco Álvares[1], Susana Lopes[1], Juan Carlos Blanco[5], Yolanda Cortés[5], Emilio García[4], Vicente Palacios[4], Helena Rio-Maior[1,2], Nuno Ferrand[1,2,6], Raquel Godinho[1,2,6]

[1]CIBIO/InBio - Centro de Investigação em Biodiversidade e Recursos Genéticos, Universidade do Porto, Campus de Vairão, 4485-661 Vairão, Portugal
[2]Departamento de Biologia, Faculdade de Ciências, Universidade do Porto, 4169-007 Porto, Portugal
[3]Research Unit of Biodiversity (UO/CSIC/PA), University of Oviedo, 33600, Mieres, Spain
[4]A.RE.NA, Asesores en Recursos Naturales, S.L, 27003 Lugo, Spain
[5]Proyecto Lobo CBC, C/ Manuela Malasaña 24, 28004 Madrid, Spain
[6]Department of Zoology, Faculty of Sciences, University of Johannesburg, Auckland Park 2006, South Africa

Table of Contents

Fig. S1: Mean posterior likelihoods and ∆K values of Structure runs	2
Fig. S2: Spatial projection of Structure results for K=2 to K=12	3
Fig. S3: Spatial projection of the results from the hierarchical Structure analysis	9
Fig. S4: BIC values for each number of clusters in the DAPC analysis	10
Fig. S5: Distribution of membership proportions and cut-off values for each cluster at K=4	11
Fig. S6: Distribution of membership proportions and cut-off values for each cluster at K=11	12
Fig. S7: Spatial overlap between the genetic groups identified at K=4 and main rivers, main roads (highways), and the Sites of Community Interest (Natura 2000 Network).	14
Fig. S8: Spatial overlap between the genetic groups identified at K=11 and main rivers, main roads (highways), and the Sites of Community Interest (Natura 2000 Network).	15
Table S1: Microsatellite loci used in this study	16
Table S2: PCR conditions for microsatellite loci used in this study.	19
Table S3: Summary of the spatial behavior information	21
Table S4: Genetic diversity and relatedness of genetic clusters and geographical genetic groups at K=4	24
Table S5: Genetic diversity and relatedness of genetic clusters and geographical genetic groups at K=11	25
Table S6: Pairwise FST matrix between geographical populations and genetic cluters at K=4	27
Table S7: Pairwise FST matrix between geographical populations and genetic clusters at K=11	28
Table S8: Pairwise Jost's D distance matrix between geographical populations and genetic clusters at K=4	29
Table S9: Pairwise Jost's D distance matrix between geographical populations and genetic clusters at K=11	30

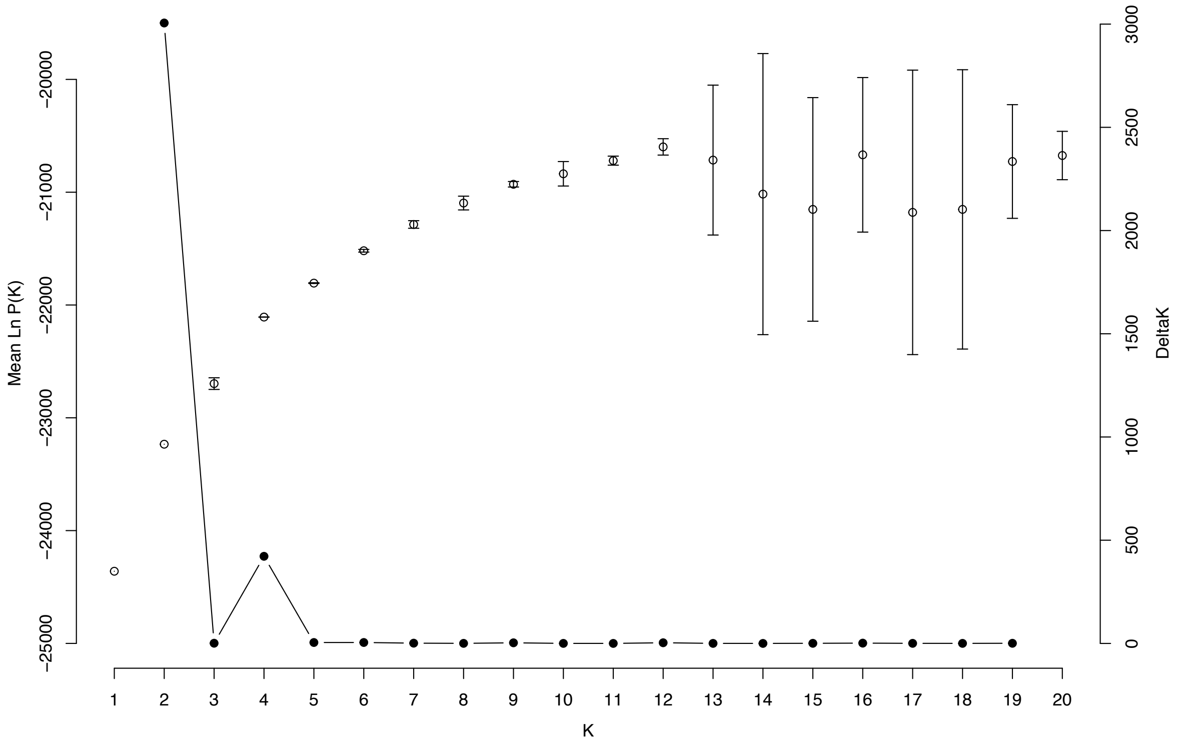

Fig. S1: Mean posterior likelihoods (open circles) and ΔK values (full circles) of Structure runs for K=1 to K=20 across 20 independent runs for each K.

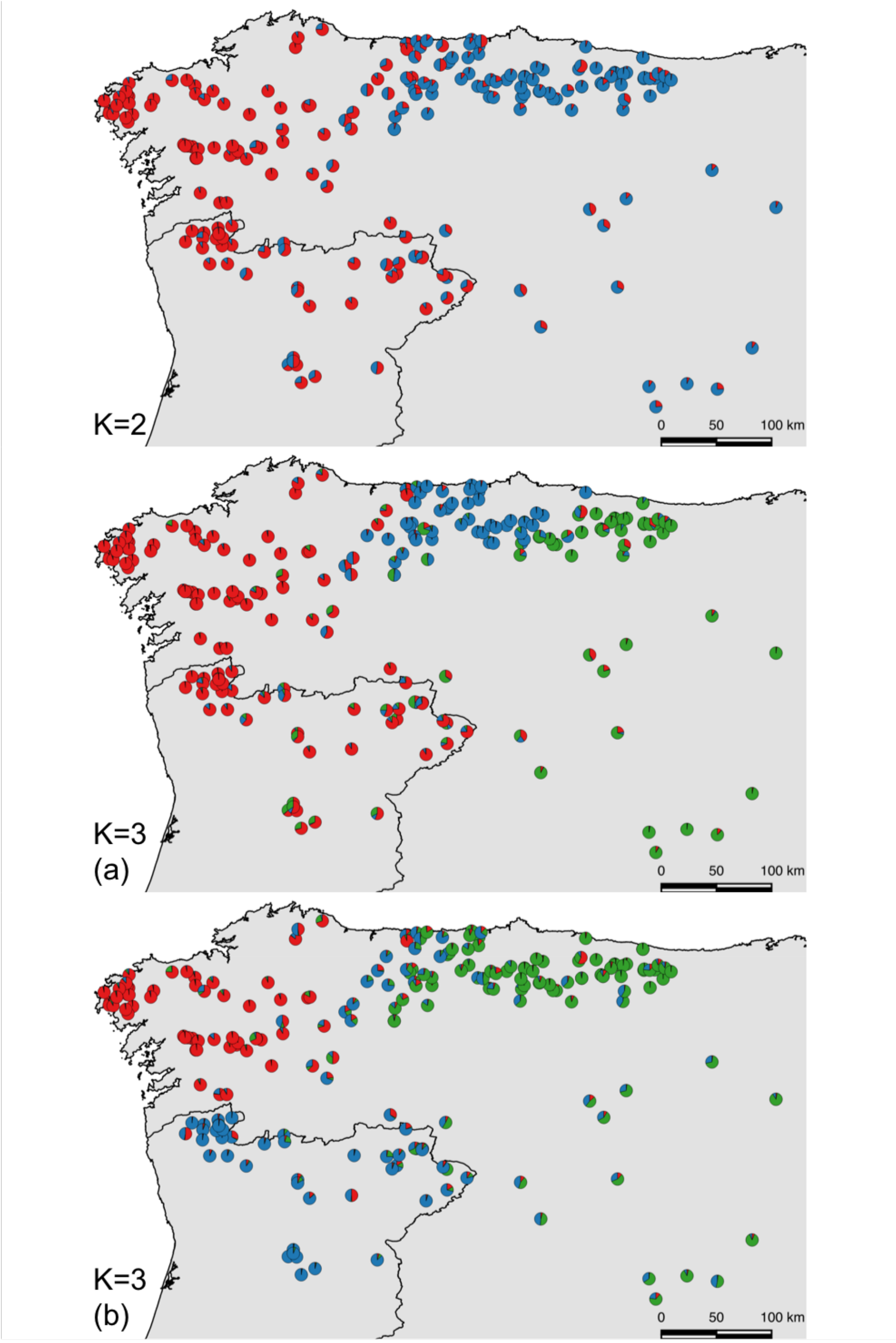

Fig. S2: **Spatial projection of Structure results for K=2 to K=12.** Each genetic cluster is represented as a different color. Individuals are represented at their sampling location as circles, with colors proportional to individual membership proportions to each cluster. Some K values presented more than one partition scheme with high posterior likelihood; in these cases, the alternatives are identified with (a), (b), ...

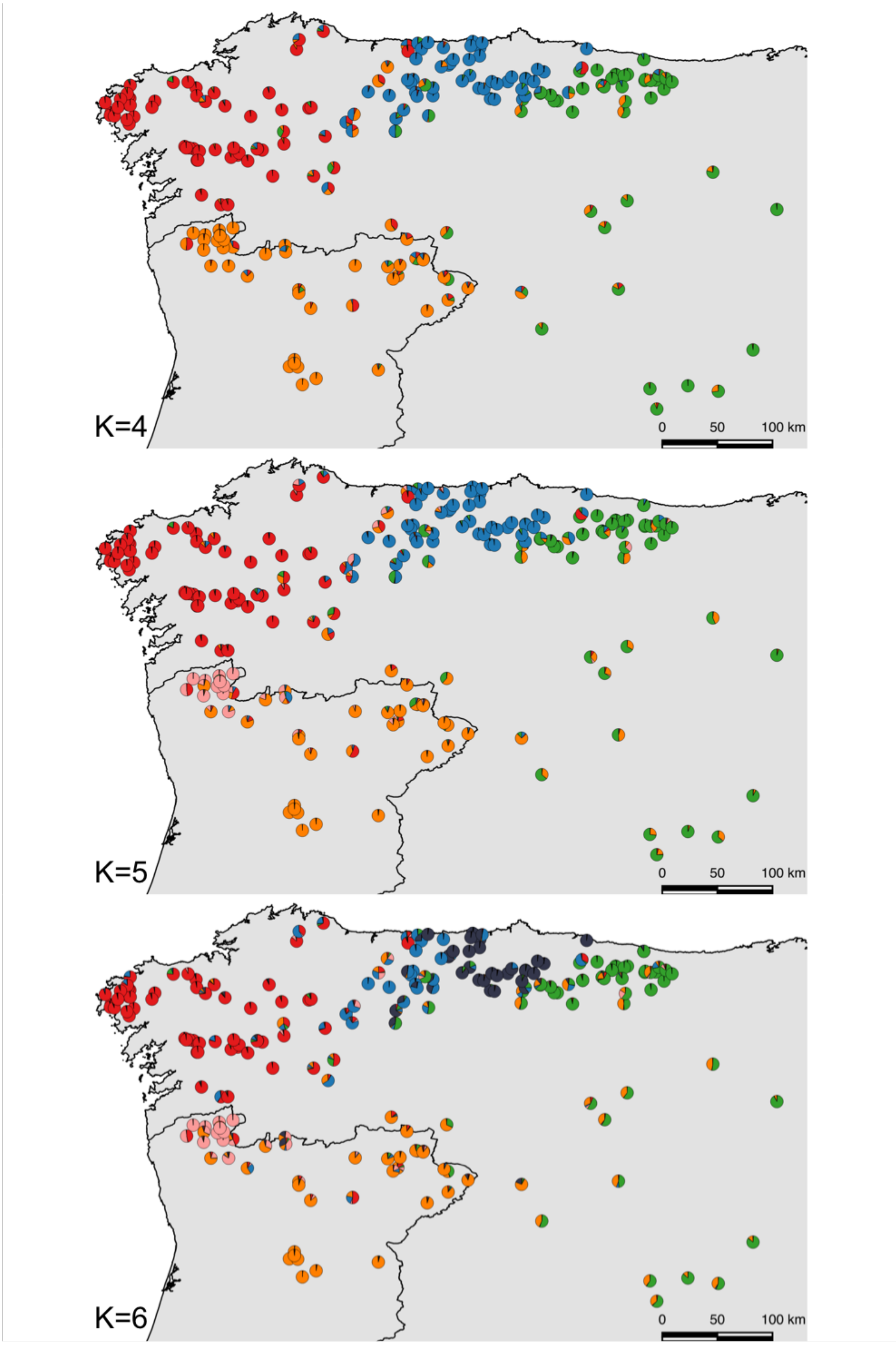

Fig. S2 (continued)

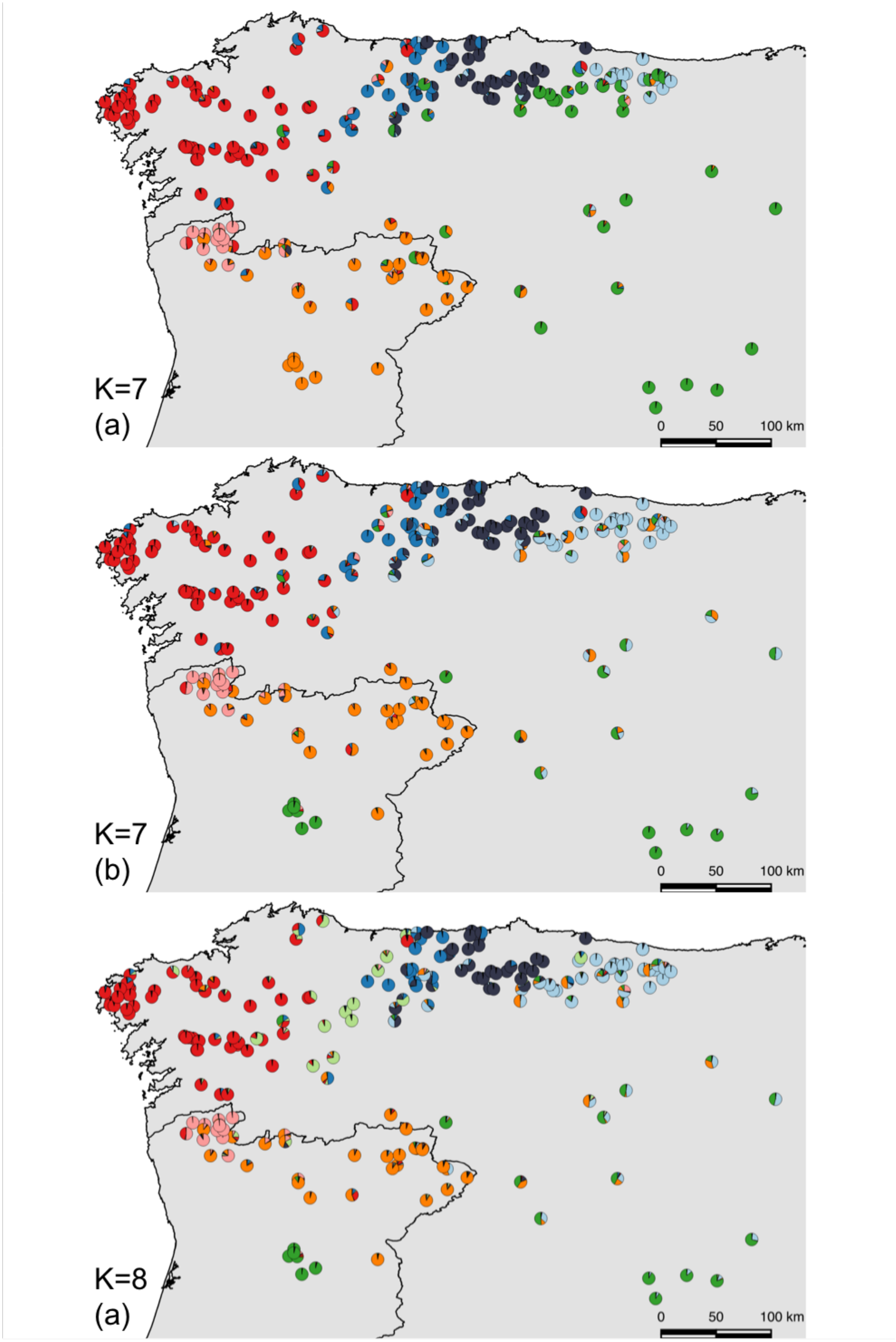

Fig. S2 (continued)

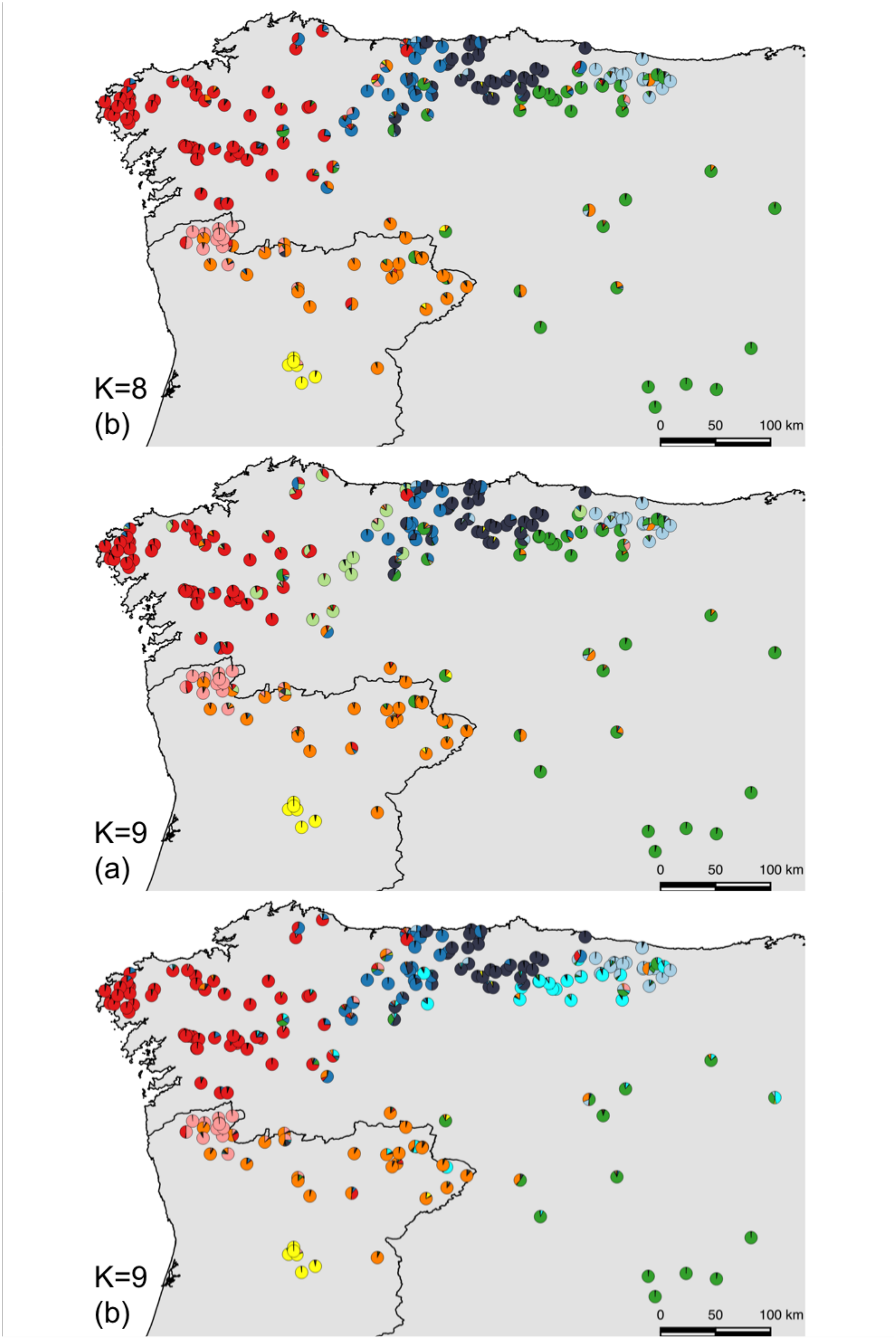

Fig. S2 (continued)

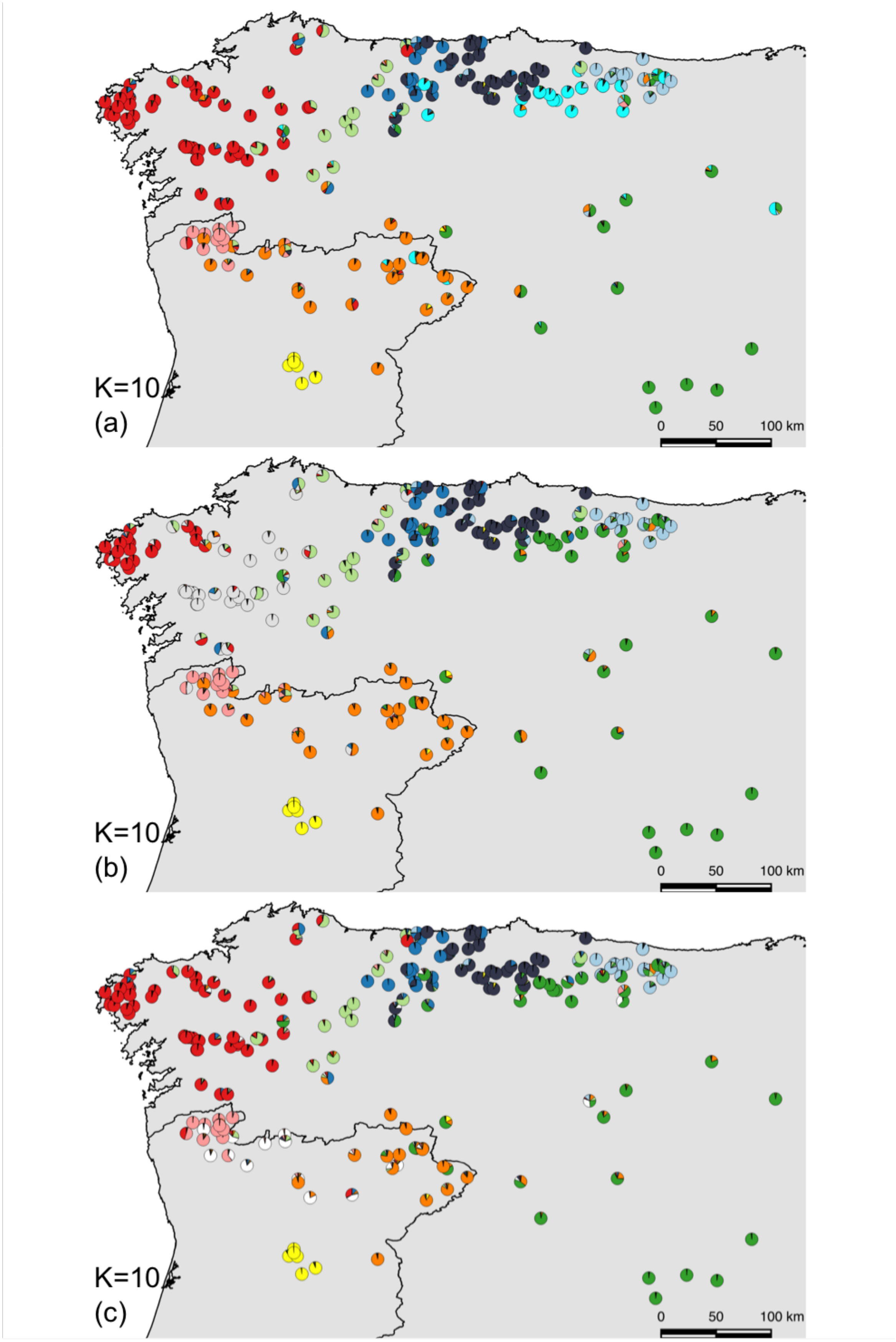

Fig. S2 (continued)

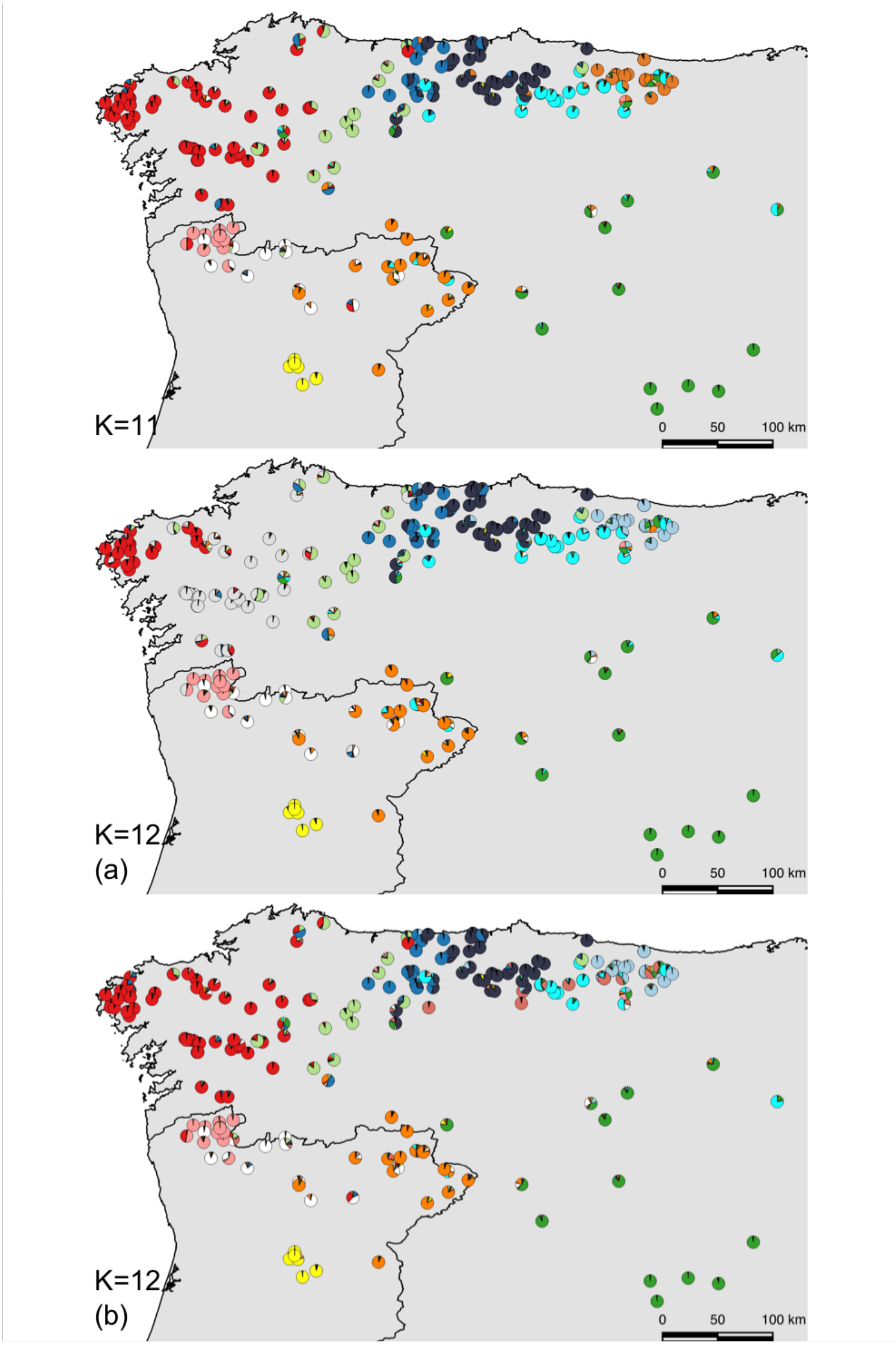

Fig. S2 (continued)

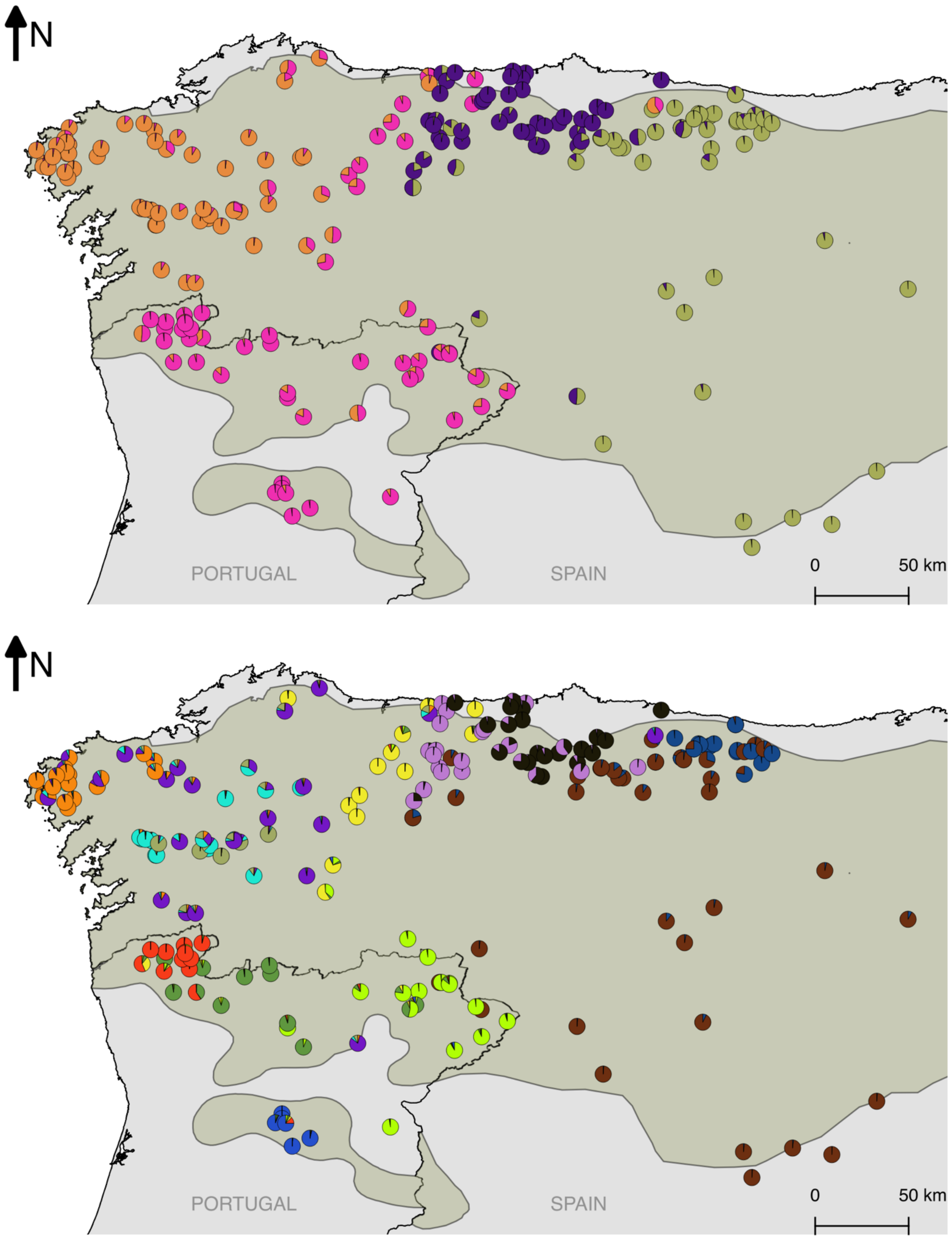

Fig. S3: Spatial projection of the results from the hierarchical Structure analysis, for a total number of clusters of four (top) and 13 (bottom). Structure was run independently for each cluster identified at K=2, and then again for each of the subsequent clusters. The grey shade represents the wolf distribution in the Iberian Peninsula.

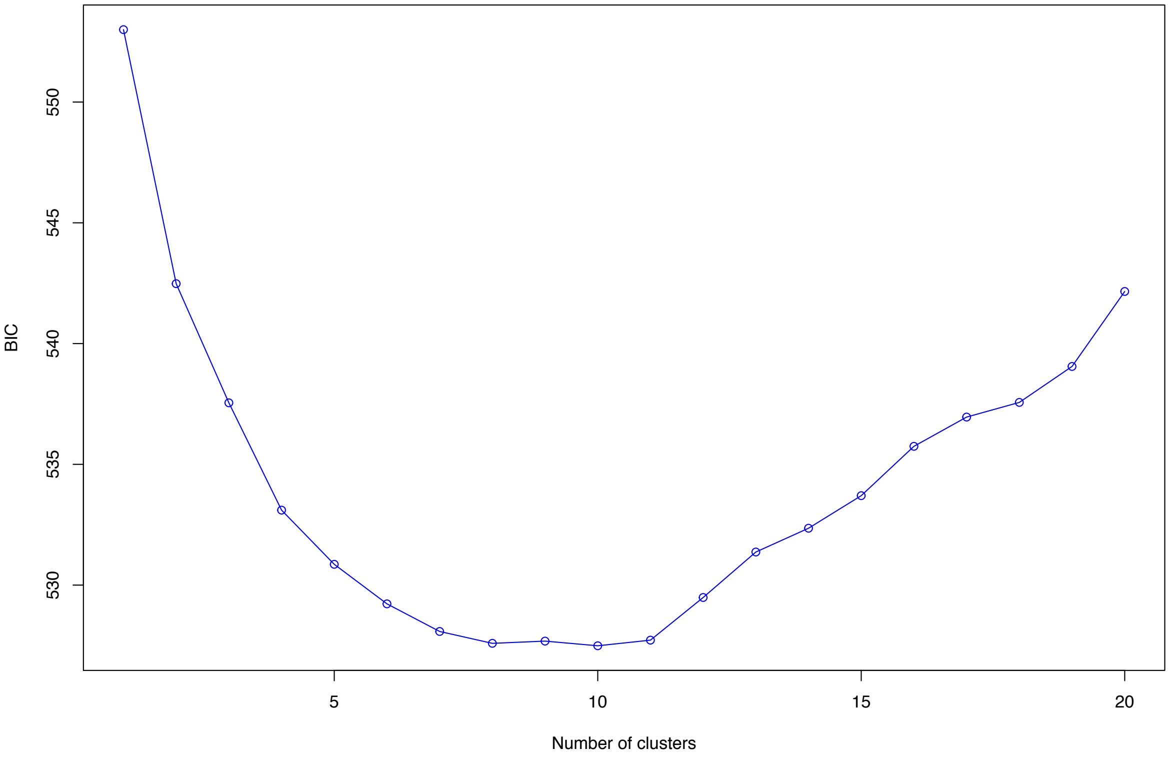

Fig. S4: Bayesian Information Criterion (BIC) values for each number of clusters, up to 20, calculated by DAPC.

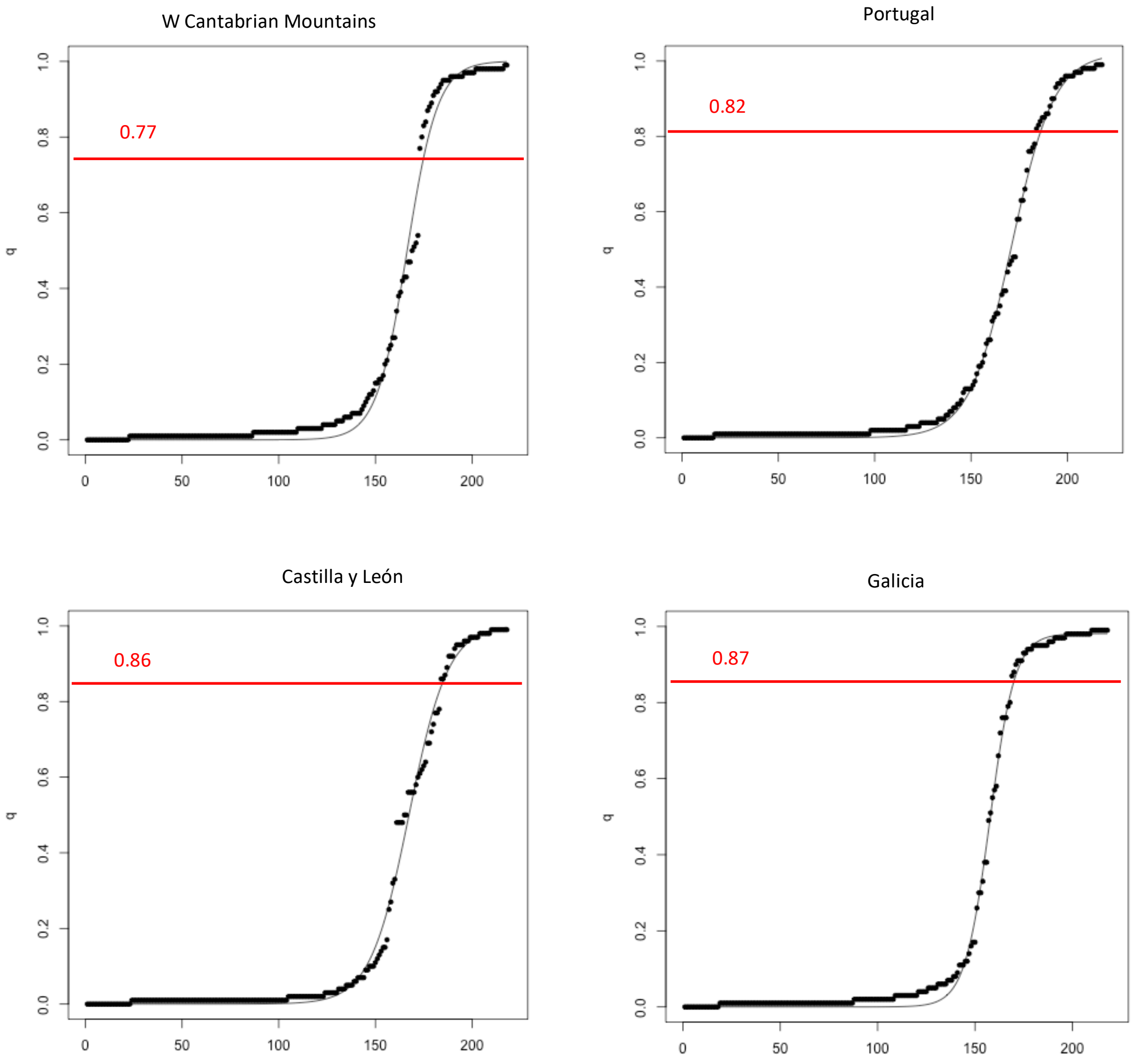

Fig. S5: Distribution of membership proportions and cut-off values for each cluster at K=4. Individuals above the cut-off value (red line) were considered as belonging to that cluster (‘non-admixed’).

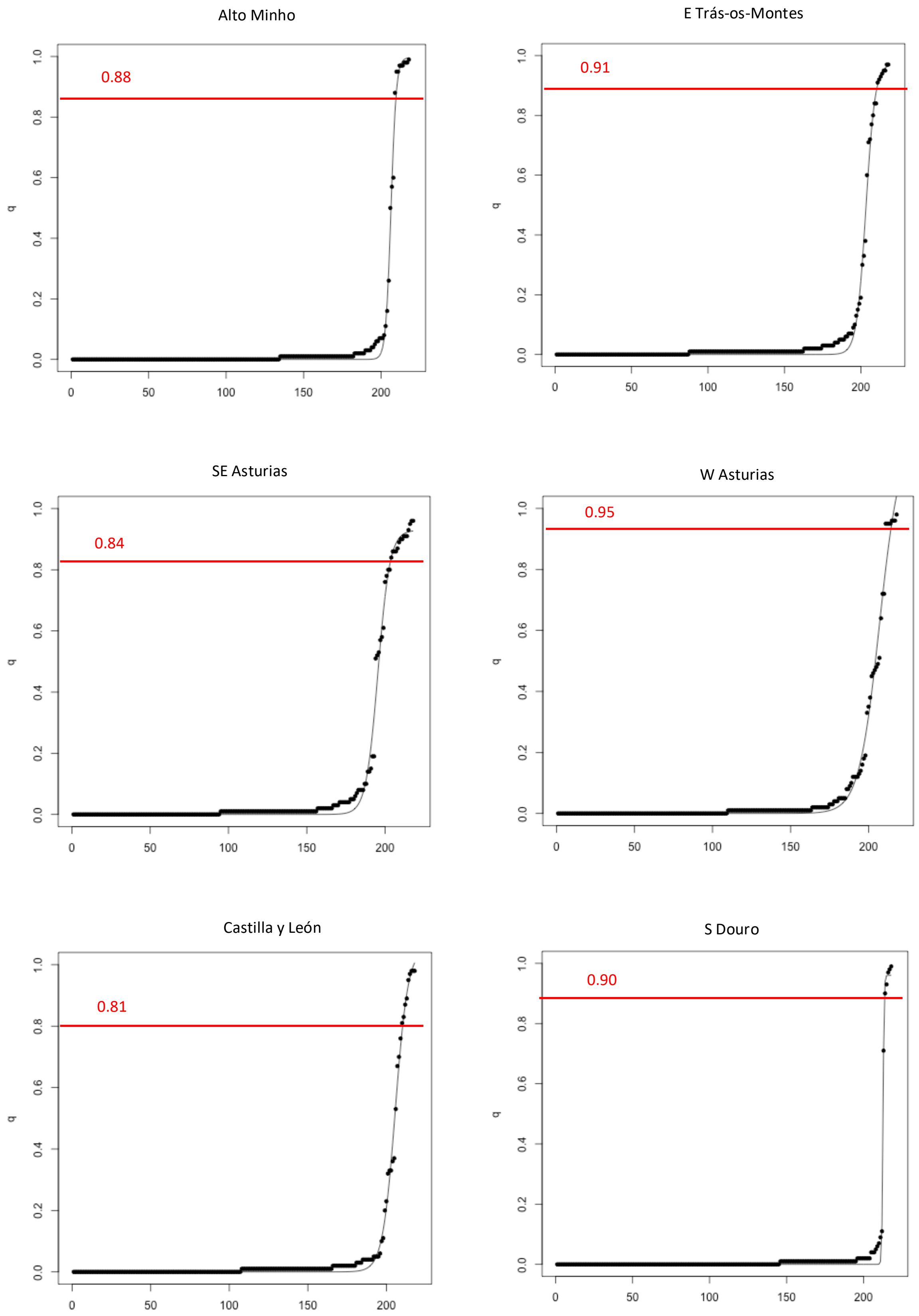

Fig. S6: Distribution of membership proportions and cut-off values for each cluster at K=11. Individuals above the cut-off value (red line) were considered as belonging to that cluster ('non-admixed').

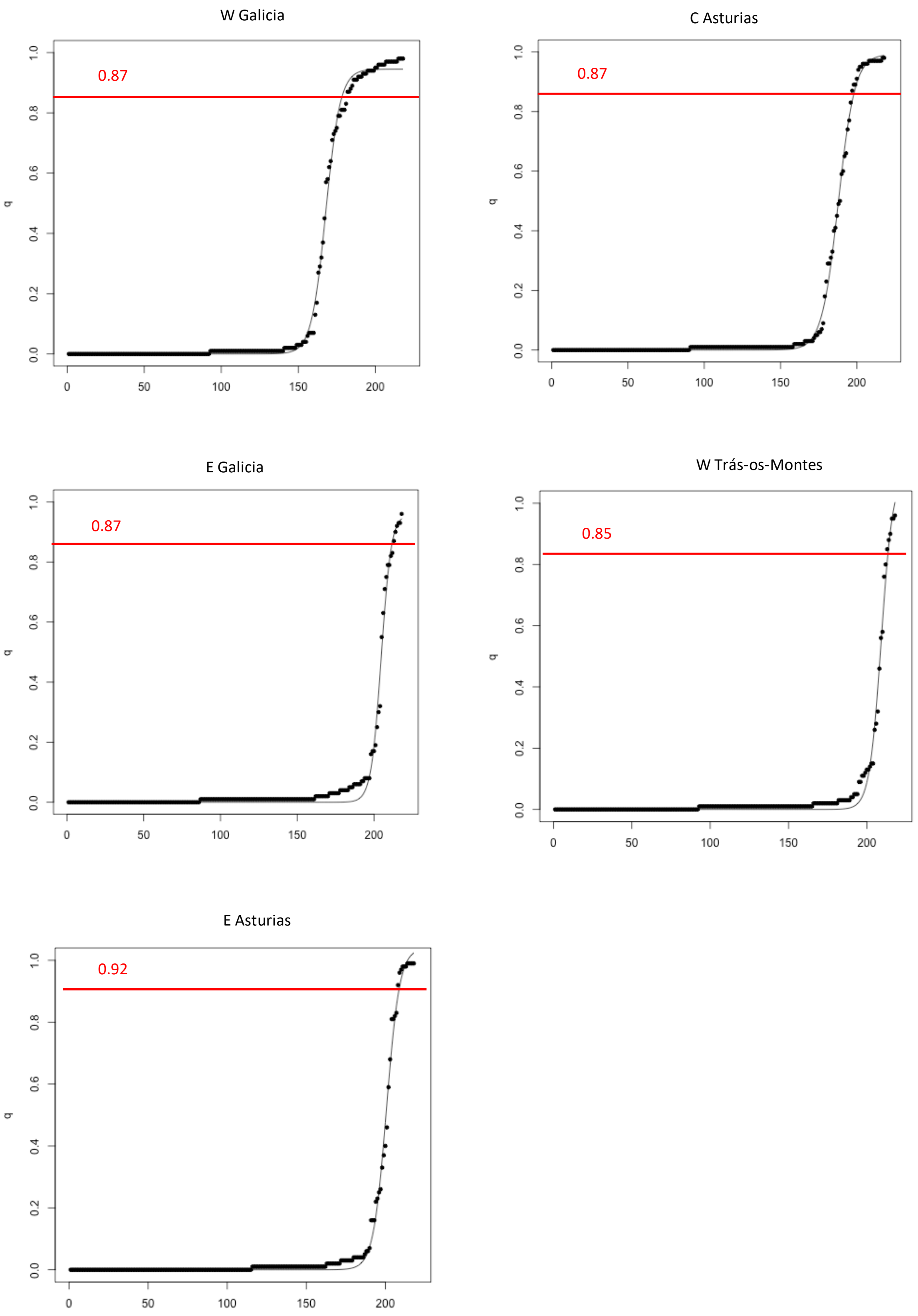

Fig. S6 (continued)

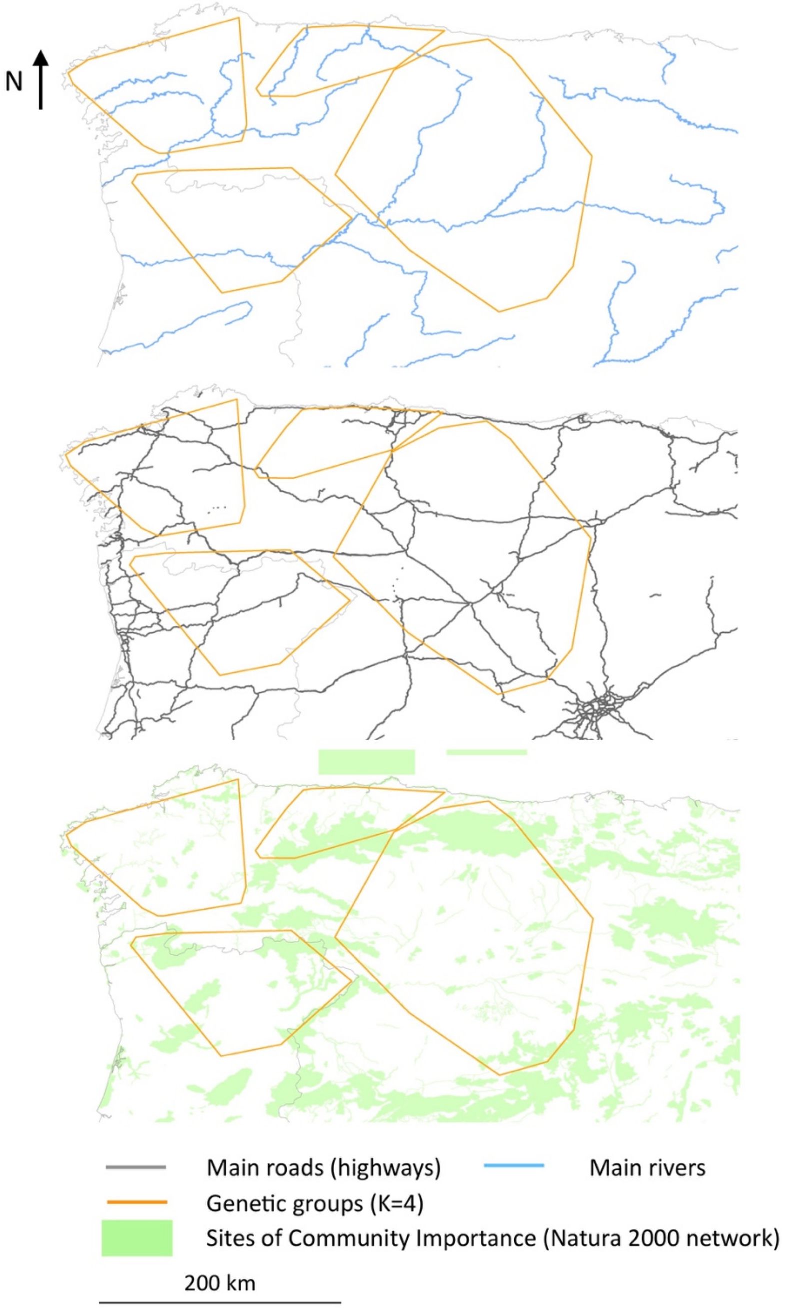

Fig. S7: Spatial overlap between the genetic groups identified at K=4 (orange polygons) and main rivers, main roads (highways), and the Sites of Community Interest (Natura 2000 Network). Estimated wolf packs in the Iberian Peninsula in 2005 (Álvares et al. 2005) are denoted with black dots and the locations of the genetic samples used in this study are shown in orange. Spatial information was extracted from Ministerio de Agricultura, Pesca y Alimentación and Centro Nacional de Información Geográfica (Spain), and Instituto Geográfico do Exército, Agência Portuguesa do Ambiente and Instituto da Conservação da Natureza e das Florestas (Portugal).

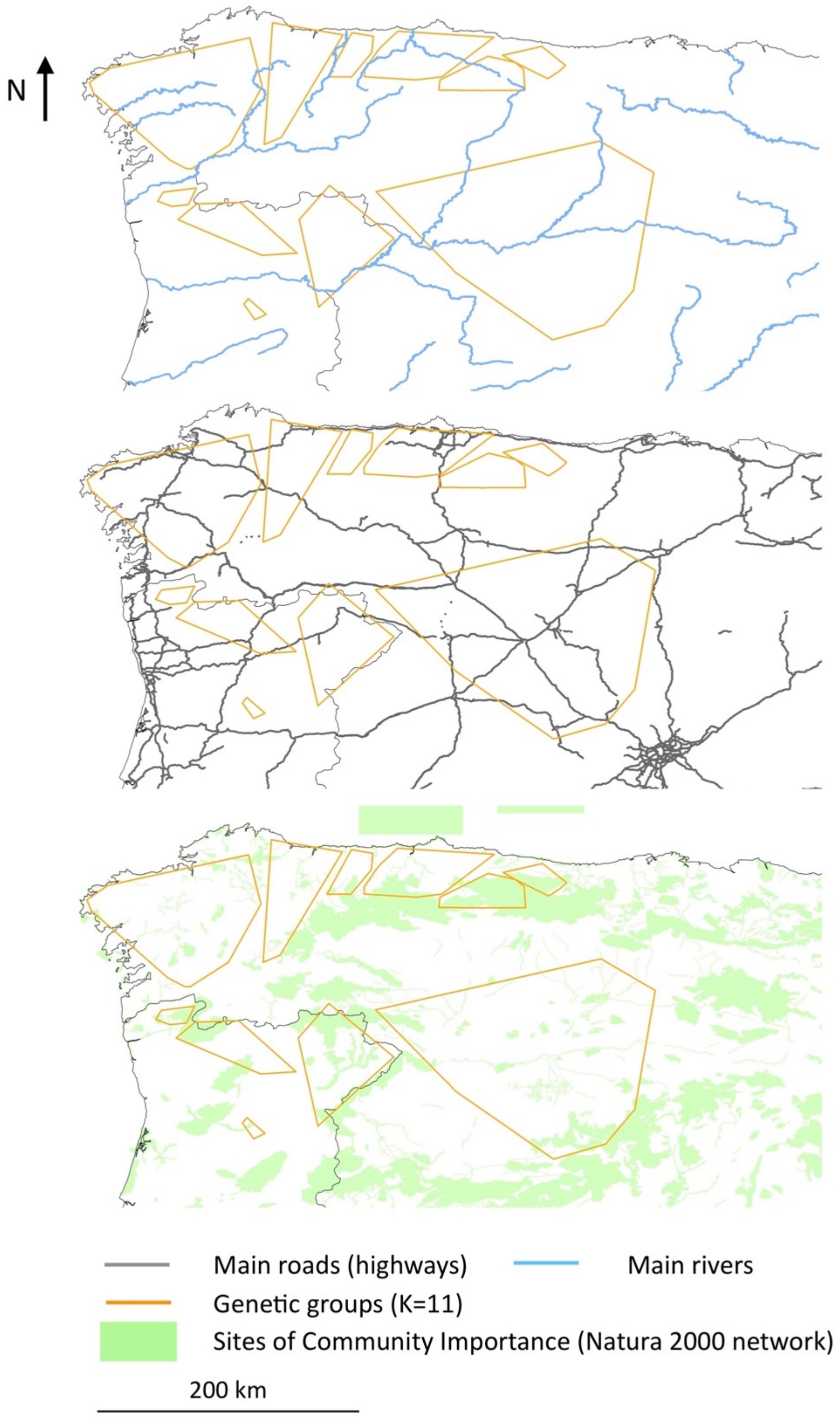

Fig. S8: Spatial overlap between the genetic groups identified at K=11 (orange polygons) and main rivers, main roads (highways), and the Sites of Community Interest (Natura 2000 Network). Estimated wolf packs in the Iberian Peninsula in 2005 (Álvares et al. 2005) are denoted with black dots and the locations of the genetic samples used in this study are shown in orange. Spatial information was extracted from Ministerio de Agricultura, Pesca y Alimentación and Centro Nacional de Información Geográfica (Spain), and Instituto Geográfico do Exército, Agência Portuguesa do Ambiente and Instituto da Conservação da Natureza e das Florestas (Portugal).

Table S1: Microsatellite loci used in this study with corresponding repeat motif, allele size range and reference.

Locus name	Repeat type	Allele Range	Reference
AHT103	Di	71-89	Holmes et al., 1995
AHT111	Di	72-92	Holmes et al., 1993
AHT121	Di	74-118	Holmes et al., 1995
AHT132	Di	170-182	by N. Holmes
AHT137	Di	128-160	Holmes et al., 1995
AHTh171	Di	216-240	Breen et al., 2001
AHTh260	Compound	229-265	Breen et al., 2001
AHTk211	Di	82-98	Lingaas et al., 1997
AHTk253	Di	280-300	Lingaas et al., 1997
C04.140	Di	132-160	Ostrander, Sprague Jr., & Rine, 1993
C08.410	Di	95-125	Ostrander, Mapa, Yee, & Rine, 1995
C08.618	Di	188-208	Ostrander et al., 1995
C09.173	Di	100-118	Ostrander et al., 1993
C09.474	Di	111-133	Ostrander et al., 1995
C13.758	Di	220-244	Mellersh et al., 1997
C14.866	Di	221-257	Mellersh et al., 1997
C20.253	Di	95-125	Ostrander et al., 1993
C20.446	Di	173-201	Ostrander et al., 1995
C22.279	Di	108-132	Ostrander et al., 1993
C27.442	Di	158-172	Ostrander et al., 1995
CPH02	Di	87-113	Fredholm & Winterø, 1995
CPH05	Di	95-131	Fredholm & Winterø, 1995
CPH09	Di	133-163	Fredholm & Winterø, 1995
CPH14	Di	185-205	Fredholm & Winterø, 1995
CXX.459	Di	141-167	Ostrander et al., 1995
Dbar2	Di	163-169	Kerns et al., 2004
FH2001	Tetra	123-155	Francisco, Langston, Mellersh, Neal, & Ostrander, 1996
FH2010	Tetra	216-240	Francisco et al., 1996
FH2054	Tetra	121-181	Francisco et al., 1996
FH2079	Tetra	246-292	Francisco et al., 1996
FH2161	Tetra	228-260	Francisco et al., 1996
FH2848	Di	224-244	Breen et al., 2001
INRA21	Di	87-103	Mariat, Kessler, Vaiman, & Panthier, 1996
INU005	Di	104-136	Finnzymes, Inc
INU030	Di	136-156	Finnzymes, Inc
INU055	Di	196-210	Finnzymes, Inc
PEZ1	Tetra	99-131	Neff et al., 1999
PEZ3	Tri	106-147	Neff et al., 1999
PEZ5	Tetra	95-119	Neff et al., 1999
REN162C04	Di	189-215	Guyon et al., 2003
REN169D01	Di	192-220	Guyon et al., 2003
REN169O18	Di	145-171	Guyon et al., 2003
REN247M23	Di	263-283	Guyon et al., 2003

REN54P11	Di	223-245	Guyon et al., 2003
REN64E19	Di	132-181	Breen et al., 2001
VWF	Hexa	138-192	Shibuya, Collins, Huang, & Johnson, 1993

References

Breen, M., Jouquand, S., Renier, C., Mellersh, C. S., Hitte, C., Holmes, N. G., … Galibert, F. 2001. Chromosome-specific single-locus FISH probes allow anchorage of an 1800-marker integrated radiation-hybrid/linkage map of the domestic dog genome to all chromosomes. *Genome Research*, *11*10, 1784–1795. doi:10.1101/gr.189401

Francisco, L. V, Langsten, A. A., Mellersh, C. S., Neal, C. L., & Ostrander, E. a. 1996. A class of highly polymorphic tetranucleotide repeats for canine genetic mapping. *Mammalian Genome*, *7*5, 359–362. doi:10.1007/s003359900104

Fredholm, M., & Winterø, a K. 1995. Variation of short tandem repeats within and between species belonging to the Canidae family. *Mammalian Genome*, *6*1, 11–8. doi:10.1007/BF00350887

Godinho, R., Llaneza, L., Blanco, J. C., Lopes, S., Álvares, F., García, E. J., … Ferrand, N. 2011. Genetic evidence for multiple events of hybridization between wolves and domestic dogs in the Iberian Peninsula. *Molecular Ecology*, *20*24, 5154–5166. doi:10.1111/j.1365-294X.2011.05345.x

Godinho, R., López-Bao, J. V., Castro, D., Llaneza, L., Lopes, S., Silva, P., & Ferrand, N. 2015. Real-time assessment of hybridization between wolves and dogs: Combining noninvasive samples with ancestry informative markers. *Molecular Ecology Resources*, *15*2, 317–328. doi:10.1111/1755-0998.12313

Guyon, R., Lorentzen, T. D., Hitte, C., Kim, L., Cadieu, E., Parker, H. G., … Ostrander, E. a. 2003. A 1-Mb resolution radiation hybrid map of the canine genome. *Proceedings of the National Academy of Sciences*, *100*9, 5296–5301. doi:10.1073/pnas.0831002100

Holmes, N. G., Dickens, H. F., Parker, H. L., Binns, M. M., Mellersh, C. S., & Sampson, J. 1995. Eighteen canine microsatellites. *Animal Genetics*, *26*2, 132a–133. doi:10.1111/j.1365-2052.1995.tb02659.x

Holmes, N. G., Humphreys, S. J., Binns, M. M., Holliman, A., Curtis, R., Mellersh, C. S., & Sampson, I. 1993. Isolation and characterization of microsatellites from the canine genome. *Animal Genetics*, *24*4, 289–292. doi:10.1111/j.1365-2052.1993.tb00313.x

Kerns, J. A., Newton, J., Berryere, T. G., Rubin, E. M., Cheng, J. F., Schmutz, S. M., & Barsh, G. S. 2004. Characterization of the dog Agouti gene and a nonagouti mutation in German Shepherd Dogs. *Mammalian Genome*, *15*10, 798–808. doi:10.1007/s00335-004-2377-1

Lingaas, F., Sørensen, A., Juneja, R. K., Johansson, S., Fredholm, M., Winterø, A. K., … Dolf, G. 1997. Towards construction of a canine linkage map: Establishment of 16 linkage groups. *Mammalian Genome*, *8*3, 218–221. doi:10.1007/s003359900393

Lynch, M., & Ritland, K. 1999. Estimation of pairwise relatedness with molecular markers. *Genetics*, *152*4, 1753–1766.

Mariat, D., Kessler, J. L., Vaiman, D., & Panthier, J. J. 1996. Polymorphism characterization of five canine microsatellites. *Animal Genetics*, *27*6, 434–435. doi:10.1111/j.1365-2052.1996.tb00514.x

Mellersh, C. S., Mellersh, C. S., Langston, a a, Langston, a a, Acland, G. M.,

Acland, G. M., … Ostrander, E. a. 1997. A linkage map of the canine genome. *Genomics*, *46*3, 326–336. doi:10.1006/geno.1997.5098
Neff, M. W., Broman, K. W., Mellersh, C. S., Ray, K., Acland, G. M., Aguirre, G. D., … Rine, J. 1999. A second-generation genetic linkage map of the domestic dog, Canis familiaris. *Genetics*, *151*2, 803–820. doi:10.1073/pnas.95.6.3048
Ostrander, E. A., Mapa, F. a., Yee, M., & Rine, J. 1995. One hundred and one new simple sequence repeat-based markers for the canine genome. *Mammalian Genome*, *6*3, 192–195. doi:10.1007/BF00293011
Ostrander, E. A., Sprague Jr., G. F., & Rine, J. 1993. Identification and characterization of dinucleotide repeat CAn markers for genetic mapping in dog. *Genomics*, *16*, 207–213.
Shibuya, H., Collins, B. K., Huang, T. H.-M., & Johnson, G. S. 1994. A polymorphic AGGAAT, tandem repeat in an intron of the canine von Willebrand factor gene. *Animal Genetics*, *25*2, 122–122. doi:10.1111/j.1365-2052.1994.tb00094.x

Table S2: PCR conditions for microsatellite loci used in this study.

Locus	Dye	Multiplex	Conditions: Temperature	Conditions: N Cycles
FH2010<br>FH2079<br>PEZ1<br>PEZ3<br>PEZ5<br>AHT132<br>C27.442	FAM<br>NED<br>FAM<br>NED<br>VIC<br>VIC<br>PET	MS1	95°C (15')	1
			95°C (30")<br>56°C (45")<br>72°C (45")	40
			60°C (30')	1
AHT103<br>AHT111<br>C04.140<br>C09.173<br>C13.758<br>C14.866<br>C20.253<br>CPH14<br>FH2001<br>VWF	NED<br>VIC<br>PET<br>NED<br>FAM<br>VIC<br>PET<br>FAM<br>FAM<br>NED	MS2	95°C (15')	1
			95°C (30")<br>56°C (45")<br>72°C (45")	40
			60°C (30')	1
C08.410<br>C08.618<br>C09.474<br>C20.446<br>CPH02<br>CPH05<br>CPH09<br>CXX459<br>FH2161<br>REN64E19	VIC<br>VIC<br>PET<br>NED<br>NED<br>FAM<br>NED<br>VIC<br>NED<br>FAM	MS3	95°C (15')	1
			95°C (30")<br>60°C (45")<br>72°C (45")	7 (-0.5°C)
			95°C (30")<br>57°C (45")<br>72°C (45")	33
			60°C (30')	1
Dbar2	FAM	-	95°C (15')	1
			95°C (30")<br>56°C (45")<br>72°C (45")	40
			60°C (15')	1

AHT121<br>AHT137<br>AHTh171<br>AHTh260<br>AHTk253<br>AHTk211<br>C22.279<br>FH2054<br>FH2848<br>INRA21<br>INU005<br>INU030<br>INU055<br>REN162C04<br>REN169D01<br>REN169O18<br>REN247M23<br>REN54P11	PET<br>VIC<br>PET<br>VIC<br>VIC<br>FAM<br>FAM<br>PET<br>NED<br>VIC<br>NED<br>NED<br>FAM<br>PET<br>VIC<br>FAM<br>PET<br>FAM	Thermo Fisher Scientific (Canine Genotypes Panel 2.1 Kit)	98°C (3')	1
			98°C (15")<br>60°C (75")<br>72°C (45")	40
			72°C (5')	1

Table S3: Summary of the spatial behavior information for 85 wolves collared in the Iberian Peninsula from 1982 to 2015. See references in methods details. Genetic groups are denoted according to Fig. 4 in the main text. "-" = Information not available/Individual not overlapping with any of the genetic groups detected.

ID	Full monitoring MCP area km^2	Period	Sampling period days	Number of locations recorded	Sex	Age yrs	Collar type	Genetic group
1	67	1982	228	-	M	>2	VHF	VIII
2	35	1982-1983	276	-	M	<2	VHF	VIII
3	65	1991	44	-	F	>2	VHF	VIII
4	91	1991-1992	165	-	F	<2	VHF	-
5	309	1996-1997	534	-	M	>2	VHF	VIII
6	288	1996-1997	534	-	F	>2	VHF	VIII
7	123	1997	169	-	M	>2	VHF	VIII
8	228	1997	159	-	M	<2	VHF	VIII
9	155	1997-1998	135	37	F	>2	VHF	VII
10	950	1997-2002	2121	242	M	<2	VHF	VII
11	530	1997-2002	1734	282	F	>2	VHF	VII
12	880	1997-2003	2101	247	F	<2	VHF	VII
13	2810	1997-2003	2129	417	F	<2	VHF	VII
14	504	1997-1999	487	-	M	<2	VHF	XI
15	1040	1998	229	47	M	>2	VHF	VII
16	132	1998-1999	119	-	M	<2	VHF	XI
17	169	1998-1999	150	-	F	>2	VHF	XI
18	695	1998-1999	563	327	M	>2	VHF	IX
19	1230	1998-2001	1029	96	F	>2	VHF	VII
20	2030	1998-2002	1645	188	M	>2	VHF	VII
21	670	1998-2003	2047	183	M	>2	VHF	VII
22	56	1999	102	89	F	<2	VHF	VII
23	23	1999	58	23	M	<2	VHF	VII
24	225	1999	142	55	M	<2	VHF	VII
25	270	1999-2000	293	56	F	>2	VHF	VII
26	398	1999-2002	784	313	M	<2	VHF	IX
27	890	2000-2003	820	135	M	>2	VHF	IX
28	640	2002-2004	638	312	F	<2	VHF	VII
29	233	2004	89	153	M	>2	GPS	VII
30	350	2004-2005	388	42	M	<2	VHF	VII
31	80	2005-2006	536	15	F	<2	VHF	VII
32	648	2005-2007	632	1853	F	>2	GPS	VII
33	238	2006	154	3484	M	>2	GPS	I
34	168	2006	52	1133	M	<2	GPS	I
35	35	2006	23	247	M	<2	GPS	I
36	570	2006	95	971	M	>2	GPS	I
37	80	2007	251	2684	F	<2	GPS	I

38	80	2007	95	1091	M	<2	GPS	I
39	189	2007	69	755	M	>2	GPS	I
40	51	2007-2008	75	861	F	>2	GPS	X
41	61	2008	103	1011	M	<2	GPS	X
42	151	2008-2010	526	5836	F	>2	GPS	X
43	391	2009	111	3377	F	>2	GPS	I
44	281	2009	344	7146	M	>2	GPS	I
45	429	2009-2010	315	10181	F	>2	GPS	I
46	831	2009-2010	241	7252	F	>2	GPS	I
47	51	2009-2010	112	2595	M	<2	GPS	I
48	191	2010-2011	129	4357	M	<2	GPS	II
49	205	2010-2011	206	5069	M	>2	GPS	VI
50	211	2010-2011	128	2498	F	<2	GPS	VI
51	1169	2010-2011	232	1948	F	>2	GPS	-
52	850	2010-2011	399	-	M	<2	GPS	XI
53	98	2010-2011	368	3005	F	>2	GPS	X
54	210	2010-2011	256	2083	M	>2	GPS	X
55	290	2011	143	3187	F	<2	GPS	I
56	235	2011	138	2989	F	<2	GPS	I
57	274	2011	139	2889	F	<2	GPS	II
58	70	2011	91	2094	F	>2	GPS	I
59	165	2011	121	2621	F	>2	GPS	I
60	1031	2011-2012	269	6433	M	>2	GPS	I
61	115	2011-2012	488	9499	F	<2	GPS	I
62	89	2011-2012	146	3486	F	>2	GPS	I
63	30	2011-2012	91	2120	M	<2	GPS	I
64	530	2011-2012	163	3920	M	>2	GPS	I-II
65	237	2011-2012	284	4866	M	<2	GPS	VI
66	911	2012	138	1214	M	<2	GPS	IX
67	291	2012	58	600	F	>2	GPS	X
68	89	2012-2013	259	2855	M	<2	GPS	I
69	32	2012-2013	71	254	M	<2	GPS	I
70	800	2012-2013	342	2977	F	>2	GPS	X
71	113	2012-2013	386	4116	F	>2	GPS	X
72	1427	2012-2014	606	6394	M	<2	GPS	X
73	55	2013	115	682	F	<2	GPS	X
74	96	2013	303	1993	F	>2	GPS	X
75	1017	2013-2014	466	4051	F	>2	GPS	X
76	225	2013-2014	401	4457	F	>2	GPS	X
77	312	2013-2014	162	7306	M	<2	GPS	I
78	410	2013-2014	307	13493	M	<2	GPS	I
79	197	2013-2014	327	13709	M	<2	GPS	I
80	314	2014	285	4792	F	>2	GPS	I
81	1725	2014	286	7605	M	<2	GPS	I-X
82	83	2012	75	962	M	<2	GPS	V
83	14	2012	59	948	F	<2	GPS	V

84	48	2014	16	315	M	>2	GPS	I
85	124	2015	43	542	M	>2	GPS	-

Table S4: Genetic diversity and relatedness of genetic clusters and geographical genetic groups at K=4. N: sample size; Na: number of alleles; AR: allelic richness rarefied to 46 samples; pAR: private allelic richness rarefied to 46 samples; Ho: observed heterozygosity; He: expected heterozygosity; F: fixation index; r: relatedness (Lynch & Ritland, 1999).

**Genetic group**		**N**	**Na**	**AR**	**pAR**	**Ho**	**He**	**F**	**r**
**W Cantabrian Mountains**	**group**	50.11 ± 0.50	4.59 ± 0.23	4.44 ± 0.22	0.24 ± 0.07	0.55 ± 0.03	0.59 ± 0.03	0.06 ± 0.02	0.082 0.072-0.088
	**cluster**	44.52 ± 0.42	4.39 ± 0.24	4.09 ± 0.20	0.25 ± 0.06	0.54 ± 0.03	0.59 ± 0.03	0.07 ± 0.02	0.092 0.084-0.100
**N Portugal**	**group**	45.85 ± 0.27	5.13 ± 0.25	5.00 ± 0.24	0.50 ± 0.10	0.57 ± 0.02	0.62 ± 0.02	0.08 ± 0.02	0.056 0.050-0.064
	**cluster**	34.02 ± 0.22	4.85 ± 0.22	4.64 ± 0.20	0.55 ± 0.10	0.57 ± 0.02	0.61 ± 0.02	0.07 ± 0.02	0.079 0.069-0.090
**Castilla y León**	**group**	52.13 ± 0.61	4.74 ± 0.27	4.56 ± 0.25	0.25 ± 0.07	0.53 ± 0.03	0.57 ± 0.03	0.07 ± 0.02	0.096 0.090-0.104
	**cluster**	33.22 ± 0.40	4.11 ± 0.24	3.92 ± 0.22	0.19 ± 0.05	0.52 ± 0.03	0.55 ± 0.03	0.06 ± 0.03	0.164 0.151-0.176
**Galicia**	**group**	56.39 ± 0.50	4.85 ± 0.26	4.55 ± 0.23	0.32 ± 0.07	0.55 ± 0.03	0.57 ± 0.03	0.03 ± 0.01	0.092 0.086-0.098
	**cluster**	47.76 ± 0.47	4.57 ± 0.26	4.08 ± 0.21	0.31 ± 0.07	0.54 ± 0.03	0.55 ± 0.03	0.02 ± 0.02	0.110 0.103-0.116

Table S5: Genetic diversity and relatedness of geographical genetic groups and genetic clusters at K=11. N: sample size; Na: number of alleles; Ne: number of effective alleles; AR: allelic richness rarefied to 10 samples for the populations, and to 6 samples for the clusters; pAR: private allelic richness rarefied to 10 samples for the populations, and to 6 samples for the clusters; Ho: observed heterozygosity; He: expected heterozygosity; F: fixation index; r: relatedness (Lynch and Ritland, 1999)

Genetic group		N	Na	AR	pAR	Ho	He	F	r Lynch & Ritland 1999
Alto Minho	group	12.78 ± 0.14	3.67 ± 0.16	2.93 ± 0.11	0.10 ± 0.03	0.53 ± 0.03	0.52 ± 0.03	-0.01 ± 0.04	0.245 0.214-0.279
	cluster	9.80 ± 0.12	2.94 ± 0.13	2.31 ± 0.09	0.09 ± 0.03	0.50 ± 0.04	0.47 ± 0.03	-0.02 ± 0.04	0.31 0.28-0.339
W Trás-os-Montes	group	10.78 ± 0.07	3.78 ± 0.16	3.22 ± 0.12	0.05 ± 0.02	0.57 ± 0.03	0.56 ± 0.03	-0.02 ± 0.03	0.074 0.048-0.102
	cluster	5.96 ± 0.03	3.02 ± 0.16	2.54 ± 0.10	0.06 ± 0.03	0.61 ± 0.04	0.51 ± 0.03	-0.21 ± 0.04	0.204 0.119-0.291
E Asturias	group	17.98 ± 0.25	3.46 ± 0.18	2.73 ± 0.12	0.03 ± 0.02	0.51 ± 0.04	0.49 ± 0.03	-0.04 ± 0.03	0.254 0.225-0.285
	cluster	10.35 ± 0.18	2.44 ± 0.13	2.07 ± 0.10	0.03 ± 0.02	0.46 ± 0.04	0.40 ± 0.03	-0.14 ± 0.04	0.445 0.397-0.494
E Trás-os-Montes	group	16.39 ± 0.15	4.26 ± 0.21	3.33 ± 0.13	0.11 ± 0.04	0.62 ± 0.03	0.59 ± 0.03	-0.05 ± 0.02	0.089 0.07-0.11
	cluster	7.65 ± 0.09	3.37 ± 0.16	2.59 ± 0.11	0.08 ± 0.03	0.61 ± 0.04	0.52 ± 0.03	-0.17 ± 0.03	0.179 0.134-0.23
SE Asturias	group	20.46 ± 0.15	4.07 ± 0.22	3.10 ± 0.14	0.06 ± 0.02	0.56 ± 0.03	0.55 ± 0.03	-0.01 ± 0.02	0.12 0.104-0.137
	cluster	14.54 ± 0.11	3.72 ± 0.20	2.59 ± 0.11	0.03 ± 0.01	0.56 ± 0.04	0.53 ± 0.03	-0.06 ± 0.03	0.157 0.131-0.183
W Asturias	group	11.67 ± 0.11	3.65 ± 0.17	3.00 ± 0.12	0.10 ± 0.04	0.58 ± 0.03	0.56 ± 0.02	-0.047 ± 0.03	0.151 0.116-0.19
	cluster	7.67 ± 0.11	3.00 ± 0.16	2.43 ± 0.09	0.10 ± 0.04	0.55 ± 0.04	0.51 ± 0.02	-0.09 ± 0.05	0.232 0.191-0.275

Table S5 continued

Genetic group		N	Na	AR	pAR	Ho	He	F	r<br>Lynch & Ritland 1999
**Castilla y León**	**group**	12.78 ± 0.27	3.70 ± 0.22	3.07 ± 0.16	0.09 ± 0.03	0.51 ± 0.03	0.54 ± 0.03	0.05 ± 0.03	0.148 0.124-0.176
	**cluster**	8.22 ± 0.17	3.20 ± 0.21	2.51 ± 0.12	0.09 ± 0.02	0.51 ± 0.03	0.51 ± 0.03	0.00 ± 0.04	0.214 0.177-0.251
**S Douro**	**group**	5.89 ± 0.05	2.87 ± 0.14	2.76 ± 0.13	0.15 ± 0.05	0.55 ± 0.04	0.48 ± 0.03	-0.14 ± 0.04	0.377 0.303-0.455
	**cluster**	4.89 ± 0.05	2.57 ± 0.10	2.31 ± 0.08	0.17 ± 0.05	0.55 ± 0.04	0.46 ± 0.02	-0.21 ± 0.04	0.399 0.316-0.501
**W Galicia**	**group**	50.67 ± 0.50	4.67 ± 0.26	3.04 ± 0.12	0.09 ± 0.02	0.54 ± 0.03	0.56 ± 0.03	0.02 ± 0.02	0.104 0.098-0.11
	**cluster**	35.26 ± 0.36	4.09 ± 0.23	2.51 ± 0.09	0.09 ± 0.03	0.53 ± 0.03	0.54 ± 0.03	0.02 ± 0.02	0.131 0.121-0.14
**C Asturias**	**group**	29.01 ± 0.30	3.89 ± 0.20	2.95 ± 0.12	0.04 ± 0.01	0.53 ± 0.03	0.56 ± 0.03	0.03 ± 0.03	0.16 0.147-0.174
	**cluster**	21.24 ± 0.24	3.46 ± 0.18	2.46 ± 0.09	0.04 ± 0.01	0.50 ± 0.03	0.53 ± 0.03	0.03 ± 0.03	0.198 0.177-0.217
**E Galicia**	**group**	10.13 ± 0.20	3.94 ± 0.21	3.31 ± 0.14	0.07 ± 0.02	0.62 ± 0.04	0.59 ± 0.03	-0.06 ± 0.03	0.106 0.073-0.147
	**cluster**	6.44 ± 0.15	3.17 ± 0.16	2.61 ± 0.11	0.06 ± 0.02	0.62 ± 0.4	0.53 ± 0.04	-0.17 ± 0.04	0.198 0.128-0.275

Table S6: Pairwise F_{ST} matrix between geographical populations below diagonal and genetic clusters above diagonal at K=4. All values were statistically significant based on 1000 permutations p<0.01

	**W Cantabrian Mountains**	**N Portugal**	**Castilla y León**	**Galicia**
**W Cantabrian Mountains**	--	0.08	0.10	0.10
**N Portugal**	0.07	--	0.12	0.08
**Castilla y León**	0.07	0.08	--	0.14
**Galicia**	0.09	0.06	0.11	--

Table S7: Pairwise F_{ST} matrix between geographical populations below diagonal and genetic clusters above diagonal at K=11. All values were statistically significant based on 1000 permutations p<0.05

	Alto Minho	E Trás-os-Montes	SE Asturias	W Asturias	Castilla Y León	S Douro	W Galicia	C Asturias	E Galicia	W Trás-os-Montes	E Asturias
**Alto Minho**	--	0.22	0.21	0.22	0.26	0.27	0.18	0.22	0.18	0.15	0.32
**E Trás-os-Montes**	0.13	--	0.14	0.16	0.16	0.20	0.13	0.17	0.11	0.13	0.26
**SE Asturias**	0.17	0.08	--	0.16	0.12	0.21	0.13	0.12	0.15	0.14	0.15
**W Asturias**	0.16	0.07	0.10	--	0.20	0.26	0.17	0.17	0.11	0.18	0.27
**Castilla y León**	0.20	0.10	0.08	0.14	--	0.23	0.17	0.18	0.17	0.19	0.21
**S Douro**	0.22	0.14	0.18	0.19	0.19	--	0.22	0.21	0.21	0.22	0.33
**W Galicia**	0.14	0.08	0.11	0.12	0.13	0.19	--	0.15	0.09	0.12	0.23
**C Asturias**	0.17	0.10	0.09	0.08	0.14	0.19	0.12	--	0.13	0.14	0.20
**E Galicia**	0.11	0.07	0.11	0.06	0.13	0.16	0.07	0.11	--	0.14	0.25
**W Trás-os-Montes**	0.07	0.03	0.09	0.08	0.12	0.17	0.07	0.09	0.06	--	0.28
**E Asturias**	0.23	0.14	0.07	0.15	0.12	0.25	0.16	0.13	0.16	0.16	--

Table S8: Pairwise Jost's D distance matrix between geographical populations below diagonal and genetic clusters above diagonal at K=4

	**Asturias**	**Portugal**	**Castilla y León**	**Galicia**
**Asturias**	--	0.14	0.14	0.16
**Portugal**	0.11	--	0.19	0.13
**Castilla y León**	0.11	0.14	--	0.20
**Galicia**	0.14	0.10	0.16	--

Table S9: Pairwise Jost's D distance matrix between geographical populations below diagonal and genetic clusters above diagonal at K=11

	**Alto Minho**	**E Trás-os-Montes**	**SE Asturias**	**W Asturias**	**Castilla Y León**	**S Douro**	**W Galicia**	**C Asturias**	**E Galicia**	**W Trás-os-Montes**	**E Asturias**
**Alto Minho**	--	0.30	0.29	0.30	0.38	0.37	0.25	0.32	0.24	0.19	0.40
**E Trás-os-Montes**	0.19	--	0.19	0.23	0.23	0.29	0.18	0.24	0.15	0.18	0.33
**SE Asturias**	0.26	0.12	--	0.23	0.16	0.31	0.18	0.16	0.22	0.20	0.17
**W Asturias**	0.24	0.12	0.15	--	0.31	0.40	0.24	0.24	0.16	0.27	0.33
**Castilla Y León**	0.32	0.16	0.11	0.23	--	0.33	0.24	0.27	0.26	0.29	0.24
**S Douro**	0.32	0.21	0.28	0.29	0.29	--	0.33	0.31	0.30	0.32	0.40
**W Galicia**	0.20	0.12	0.16	0.18	0.20	0.29	--	0.21	0.13	0.17	0.30
**C Asturias**	0.26	0.16	0.13	0.12	0.21	0.28	0.18	--	0.19	0.20	0.24
**E Galicia**	0.17	0.11	0.18	0.10	0.22	0.26	0.10	0.17	--	0.21	0.32
**W Trás-os-Montes**	0.10	0.05	0.14	0.13	0.19	0.25	0.10	0.14	0.10	--	0.34
**E Asturias**	0.31	0.20	0.08	0.20	0.16	0.34	0.23	0.17	0.22	0.22	--
